# Acetyl-CoA carboxylase 1–dependent lipogenesis promotes autophagy downstream of AMPK

**DOI:** 10.1074/jbc.RA118.007020

**Published:** 2019-06-17

**Authors:** Angelina S. Gross, Andreas Zimmermann, Tobias Pendl, Sabrina Schroeder, Hannes Schoenlechner, Oskar Knittelfelder, Laura Lamplmayr, Ana Santiso, Andreas Aufschnaiter, Daniel Waltenstorfer, Sandra Ortonobes Lara, Sarah Stryeck, Christina Kast, Christoph Ruckenstuhl, Sebastian J. Hofer, Birgit Michelitsch, Martina Woelflingseder, Rolf Müller, Didac Carmona-Gutierrez, Tobias Madl, Sabrina Büttner, Kai-Uwe Fröhlich, Andrej Shevchenko, Tobias Eisenberg

**Affiliations:** ‡ Institute of Molecular Biosciences, NAWI Graz, University of Graz, 8010 Graz, Austria; § Central Lab Gracia, NAWI Graz, University of Graz, 8010 Graz, Austria; ¶ BioTechMed-Graz, 8010 Graz, Austria; ‖ Max Planck Institute of Molecular Cell Biology and Genetics, 01307 Dresden, Germany; ** Department of Molecular Biosciences, Wenner-Gren Institute, Stockholm University, 114 19 Stockholm, Sweden; ‡‡ Gottfried Schatz Research Center for Cell Signaling, Metabolism, and Aging, Institute of Molecular Biology and Biochemistry, Medical University of Graz, 8036 Graz, Austria; §§ Division of Plastic, Aesthetic, and Reconstructive Surgery, Department of Surgery, Medical University of Graz, 8036 Graz, Austria; ¶¶ Helmholtz Institute for Pharmaceutical Research Saarland, 66123 Saarbrücken, Germany

**Keywords:** acetyl coenzyme A (acetyl-CoA), acetate, aging, autophagy, lipid metabolism, yeast, Acc1, acetyl-CoA carboxylase 1, AMPK, oleate, Snf1, lipogenesis

## Abstract

Autophagy, a membrane-dependent catabolic process, ensures survival of aging cells and depends on the cellular energetic status. Acetyl-CoA carboxylase 1 (Acc1) connects central energy metabolism to lipid biosynthesis and is rate-limiting for the *de novo* synthesis of lipids. However, it is unclear how *de novo* lipogenesis and its metabolic consequences affect autophagic activity. Here, we show that in aging yeast, autophagy levels highly depend on the activity of Acc1. Constitutively active Acc1 (*acc1^S/A^*) or a deletion of the Acc1 negative regulator, Snf1 (yeast AMPK), shows elevated autophagy levels, which can be reversed by the Acc1 inhibitor soraphen A. Vice versa, pharmacological inhibition of Acc1 drastically reduces cell survival and results in the accumulation of Atg8-positive structures at the vacuolar membrane, suggesting late defects in the autophagic cascade. As expected, *acc1^S/A^* cells exhibit a reduction in acetate/acetyl-CoA availability along with elevated cellular lipid content. However, concomitant administration of acetate fails to fully revert the increase in autophagy exerted by *acc1^S/A^*. Instead, administration of oleate, while mimicking constitutively active Acc1 in WT cells, alleviates the vacuolar fusion defects induced by Acc1 inhibition. Our results argue for a largely lipid-dependent process of autophagy regulation downstream of Acc1. We present a versatile genetic model to investigate the complex relationship between acetate metabolism, lipid homeostasis, and autophagy and propose Acc1-dependent lipogenesis as a fundamental metabolic path downstream of Snf1 to maintain autophagy and survival during cellular aging.

## Introduction

As age-associated diseases compromise human health span and are becoming increasingly prevalent, a better understanding of the aging process is urgently needed (1, 2). Autophagy, an intracellular degradation mechanism, is crucial for healthy aging and ensures cellular and organismal homeostasis (3–5). Macroautophagy (hereafter termed autophagy) mediates the incorporation of bulk cellular material into double-membrane vesicles, termed “autophagosomes.” The engulfed material, ranging from specific proteins to whole organelles, is then targeted to lysosomes for degradation. Therefore, autophagy ensures proteostasis as well as cellular homeostasis (6), whereas, in turn, loss of autophagy accelerates aging and favors the development of disease in many organisms (3, 4). Autophagy is under strict metabolic control (7, 8) and thus may also depend on a balanced lipid metabolism; in fact, the function of diverse classes of lipids in the regulation and execution of autophagy is an emerging research topic (9). However, to what extent *de novo* lipogenesis contributes to the control of autophagy in aging remains largely elusive.

Eukaryotic model organisms, such as the baker's yeast *Saccharomyces cerevisiae*, have been instrumental in deciphering mechanisms of cellular aging and autophagy (10–12). For instance, one of the aging paradigms in yeast, chronological life span (*i.e.* the survival time of a culture in the stationary, post-mitotic phase), serves as a model for post-mitotic aging in human cells and recapitulates the cytoprotective function of autophagy in higher organisms (13, 14), yet the importance of maintaining lipid homeostasis for cell survival and autophagy during chronological aging has hardly been addressed (15). A comprehensive understanding of yeast lipid metabolism is available (16, 17). Observations in lipid droplet (LD)^6^-deficient yeast (*i.e.* yeast unable to synthesize the major neutral lipids) suggest an important role of LDs during the acute induction of autophagy after nitrogen starvation (18, 19). However, a direct requirement of LDs for autophagy has been questioned, because LD-deficient yeast cells still induce autophagy upon rapamycin treatment (20). LD-deficient yeast also displays functional autophagy after nitrogen starvation when combined with a concomitant reduction of *de novo* fatty acid (FA) synthesis, withdrawal of inositol, or restoration of phospholipid (PL) composition by deletion of the transcriptional repressor *OPI1* (21, 22). Velázquez *et al.* (21) therefore proposed that free fatty acid (FFA)-induced ER stress limits nitrogen starvation–induced autophagy of yeast cells lacking LDs. Thus, the ability to buffer FFAs through triglyceride (TG) synthesis and storage into LDs may represent the prime function of LDs in the control of autophagy. Overall, these studies suggest that LDs regulate autophagy through balancing the cellular lipidome rather than by a direct action of TGs.

Cytosolic acetyl-CoA carboxylase (Acc1) activity is essential for cell growth in yeast (23). Acc1 catalyzes the initial and rate-limiting step of *de novo* FA synthesis by producing malonyl-CoA through carboxylation of acetyl-CoA. This activity is controlled by the glucose-sensing kinase Snf1, the homolog of the mammalian AMP-activated kinase (AMPK), which inhibits Acc1 by phosphorylation of Ser-659 and Ser-1157 (24–26). Accordingly, yeast cells carrying a constitutively active Acc1 mutant with a serine 1157-to-alanine mutation (hereafter referred to as *acc1^S/A^*) display increased neutral lipid levels. This increase can be further enhanced by additional mutation of serine 659 to alanine. Thus, the *acc1-S1157A* mutation partly uncouples Acc1 from the control by AMPK, allowing for the investigation of specific Acc1-dependent effects without interfering with the many other targets of AMPK (24). Acute inhibition of Acc1 delays cell growth and proliferation, whereas it depletes intracellular lipid stores. Interestingly, LDs (i) increase in number and size when yeast enters stationary phase (24, 27), (ii) become gradually degraded in an age-dependent manner through an autophagy-dependent process termed “lipophagy” (27–30), and (iii) may provide lipid building blocks for the production of membranes when cells re-enter the cell cycle (31). However, it has not been formally addressed whether the increased production or accumulation of LDs upon entry into stationary phase is also required for cell survival during post-mitotic aging.

We have previously shown that impaired mitochondrial utilization of acetate due to deletion of the mitochondrial CoA-transferase *ACH1* causes excess secretion of acetate and up-regulation of acetyl-CoA synthetase 2 (Acs2)-dependent hyperacetylation of histones (32). This metabolic shift of acetate toward the nucleo-cytosolic pathway of acetyl-CoA synthesis led to transcriptional defects of autophagy-related genes (such as *ATG7*) with a block in autophagy and loss of survival of chronologically aging yeast. This block of autophagy could be overcome by limiting nucleo-cytosolic acetyl-CoA production through depletion of Acs2, which also restored survival of aging cells (32). Interestingly, the pathways of acetyl-CoA generation from acetate, which is relevant for histone acetylation, and the utilization of acetyl-CoA for *de novo* lipogenesis appear metabolically related (33). However, how acetyl-CoA consumption by *de novo* lipogenesis affects acetate metabolism, autophagy, and cell survival has not been investigated.

In the present study, we asked whether *de novo* FA biosynthesis is important for the ability of cells to maintain autophagic flux and survival during aging. We demonstrate that the rate-limiting step of FA biosynthesis catalyzed by Acc1 is crucial for the regulation of autophagy and survival in chronologically aging yeast. Our data show that regulation of autophagy by Acc1 depends on a combination of metabolic consequences that involve alterations in both acetate (upstream of Acc1) and lipid (downstream of Acc1) metabolism.

## Results

### Acc1 activity controls autophagy in aging yeast

**Figure 1.**
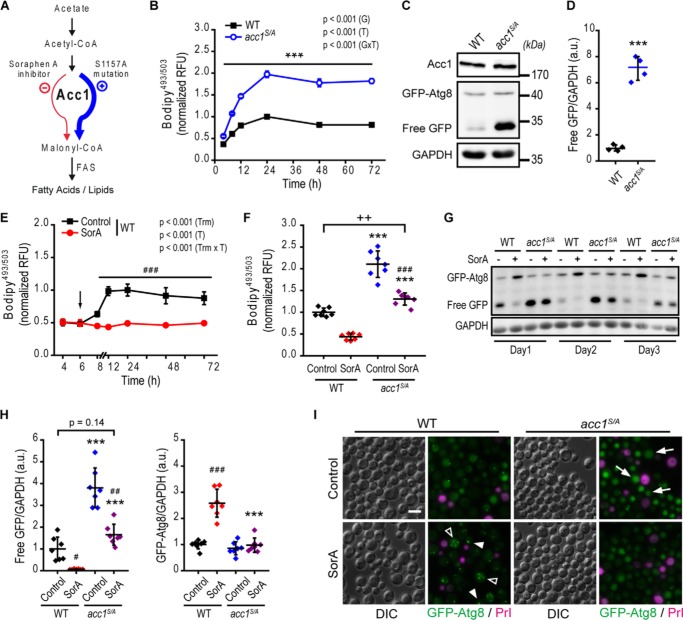
**Acc1 activity correlates with autophagy levels.** WT and *acc1-S1157A* mutant (*acc1^S/A^*) yeast cells were aged until the indicated time points on 2% glucose minimal medium. SorA or the solvent DMSO was applied 6 h after inoculation where indicated. *A*, *schematic overview* of the Acc1-regulated metabolic pathway. Acc1 activity can be modulated by SorA treatment (inhibition, *red color*) or the S1157A point mutation (activation, *blue color*). *B*, flow cytometric quantification of neutral lipids after BODIPY staining in a time course experiment. Relative fluorescence units were normalized to the WT at 24 h (*n* = 4). *C* and *D*, representative immunoblots (*C*) and quantification of free GFP/GAPDH (*D*), indicating autophagic flux (*GFP liberation assay*) of GFP-Atg8–expressing cells after 2 days of aging. Blots were probed with Acc1-, GFP-, or GAPDH-specific antibodies, and immunoblot signals were normalized to the WT. (*n* = 4). *E* and *F*, flow cytometric quantification of neutral lipids after BODIPY staining in a time course experiment (*E*) or after 24 h of incubation (day 1 of aging) (*F*). The *arrow* in *E* indicates the time of SorA application. Relative fluorescence units were normalized to the WT control at 24 h (*n* = 4 in *E*; *n* = 7 in *F*). *G* and *H*, representative immunoblots (*G*) and densitometric quantification of free GFP/GAPDH (*H*, *left*) or full-length GFP-Atg8/GAPDH levels (*H*, *right*) of GFP-Atg8–expressing cells at the indicated age (*G*) or after 2 days of aging (*H*). (*n* = 7). *I*, representative micrographs of 3-day-old cells according to *G* and *H*, demonstrating vacuolar localization of GFP indicative of autophagy (*arrows* show examples of autophagic cells) or punctate structures of GFP-Atg8. The *arrowheads* depict examples of cells with presumably enlarged pre-autophagosomal structures (1 fluorescence dot/cell), whereas *open arrowheads* show cells with accumulated autophagosomes (≥2 fluorescence puncta). Staining with PrI served to exclude dead cells from analysis. *Bar*, 5 μm. *Dot plots* show all data points along with the mean (*line*) ± S.D. (*error bar*). *Line graphs* show the mean ± S.D. *p* values indicate main effects (*G*, genotype; *T*, time; *GxT*, interaction between genotype and time) of a two-way mixed-model ANOVA, with time as repeated measures in *B* and *E*, Welch's *t* test in *D*, Welch's ANOVA post hoc Games–Howell in *H*, and ANOVA post hoc Tukey test in *F*. ***, *p* < 0.001 (compared with the respective WT); ##, *p* < 0.01; ###, *p* < 0.001 (compared with the respective untreated control, *Control*). *FAS*, fatty acid synthase; *RFU*, relative fluorescence units; *a.u.*, arbitrary units.

To address the potential role of *de novo* lipogenesis in the regulation of acetate/acetyl-CoA availability and autophagy, we decided to target the rate-limiting enzyme of FA biosynthesis, Acc1 (Fig. 1*A*). For this purpose, we used the *acc1^S/A^* mutant, which expresses constitutively active Acc1 due to S1157A mutation (24). In agreement with previously published observations (24, 25), *acc1^S/A^* cells displayed increased neutral lipid levels compared with WT cells (Fig. 1*B*), as quantified by flow cytometry after staining with the fluorescent neutral lipid dye BODIPY^TM^ 493/503 (34), hereafter referred to as BODIPY. We hypothesized that enhanced *de novo* lipogenesis in the *acc1^S/A^* mutant entails metabolic consequences that stimulate autophagy. In fact, *acc1^S/A^* mutation was sufficient to strongly induce autophagy after 2 days of chronological aging as monitored by quantifying immunoblotting-detectable free GFP (Fig. 1, *C* and *D*). This so-called “GFP liberation assay” makes use of yeast strains carrying an N-terminally GFP-tagged version of the autophagy machinery protein Atg8. Upon autophagic delivery of the fusion protein to the vacuole, Atg8 is rapidly degraded, whereas GFP remains more stable, and thus, increased levels of GFP are indicative of efficient autophagic flux (35, 36).

We then tested whether the inhibition of Acc1 showed opposite effects by applying the allosteric Acc1 inhibitor soraphen A (SorA) (37). Because Acc1 is essential for growth, SorA was applied to yeast cells in such a way that it did not affect cell growth during logarithmic phase, ensuring optical densities comparable with those under control conditions upon entry into stationary phase (Fig. S1*A*). Under these conditions, the neutral lipid content of SorA-treated cells, which remained constant throughout all tested time points, was decreased compared with untreated controls (Fig. 1*E*), confirming a reduction of Acc1-dependent lipogenesis. Thus, the addition of SorA completely prevented the increase of the neutral lipid content during transition from logarithmic growth to the stationary post-mitotic phase. Microscopic analysis and flow cytometric quantification of BODIPY-stained stationary cultures confirmed the reduction of LDs after SorA treatment in WT cells, whereas the constitutively active mutant of Acc1 (*acc1^S/A^*) retained neutral lipid levels comparable with the untreated WT controls (Fig. 1*F* and Fig. S1*B*).

Next, we explored the effects of SorA treatment on autophagy. Strikingly, SorA efficiently blocked autophagic flux of WT cells during aging, as monitored via the GFP liberation assay (Fig. 1, *G* and *H*). In WT cells, SorA treatment showed almost no free GFP generation (Fig. 1*H*, *left*) and accumulated GFP-Atg8 (Fig. 1*H*, *right*), whereas in the *acc1^S/A^* mutant, free GFP levels were maintained almost comparable with untreated WT cells. In line with this, microscopic analysis of GFP-Atg8 revealed vacuolar localization of GFP in a substantial fraction of 2-day-old WT cells, whereas after SorA treatment, the GFP signal appeared as punctate structures (presumably at least in part autophagosomes), indicating a block of autophagy that prevented the delivery of GFP-Atg8 to the vacuole (Fig. 1*I*). In contrast, SorA-treated *acc1^S/A^* mutant cells retained vacuolar GFP positivity and lacked accumulation of punctate GFP structures (Fig. 1*I*).

The strains used for autophagy observation expressed GFP-tagged Atg8 under control of the endogenous *ATG8* promoter, and thus the overall increase in GFP signals may also reflect induction of Atg8 expression, which is often associated with autophagy (38). To exclude the possibility that the strong increase in free GFP upon Acc1 activation was simply a result of the induction of endogenous Atg8 expression, we generated a fusion construct under the control of a copper-inducible promotor (*pCUP*). Using varying copper concentrations supplemented to the culture medium, we verified that in these strains GFP-Atg8 was controlled by copper addition (Fig. S1*C*). Importantly, also these strains produced more free GFP as a result of the *acc1^S/A^* mutation and displayed diminished levels of free GFP along with an accumulation of GFP-Atg8 punctate structures upon SorA treatment (Fig. S1, *D–F*).

We corroborated our data obtained with the GFP liberation assay by means of a second approach, the alkaline phosphatase assay. This assay measures autophagic flux via biochemical quantification of a cytosolically expressed form of the alkaline phosphatase Pho8 (Pho8ΔN60). Pho8ΔN60 only becomes activated upon autophagic delivery to and proteolytic processing within the vacuole (39). In accordance with our results above, *acc1^S/A^* mutant cells exhibited elevated Pho8ΔN60 activity compared with the WT, whereas SorA blocked autophagic activity almost completely in WT cells and reduced it to the levels of the WT control in the *acc1^S/A^* mutant condition (Fig. S1*G*). Altogether, our data demonstrate that the constitutively active *acc1^S/A^* mutant promotes autophagic flux.

### Acc1 drives autophagic flux downstream of yeast AMPK

**Figure 2.**
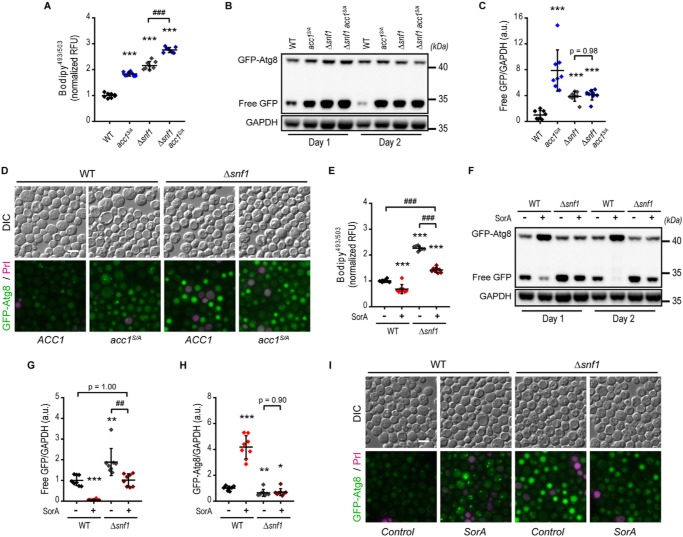
**Acc1 mediates autophagy regulation by AMPK under chronological aging conditions.** WT or yeast AMPKα subunit *SNF1*-deleted cells (Δ*snf1*) combined with or without the *acc1-S1157A mutation* (*acc1^S/A^*) were aged for 2 days on inositol-supplemented 2% glucose minimal medium. SorA or the solvent DMSO was applied to cultures 6 h after inoculation where indicated. *A*, flow cytometric quantification of neutral lipids after BODIPY staining. Relative fluorescence units were normalized to the WT (*n* = 8). *B* and *C*, representative immunoblots (*B*) and densitometric quantification of free GFP/GAPDH (*C*) of GFP-Atg8–expressing cells. (*n* = 8). *D*, representative micrographs of GFP-Atg8–expressing cells according to *A–C* demonstrating vacuolar localization of GFP indicative of autophagy. Staining with PrI served to exclude dead cells from analysis. *Bar*, 5 μm. *E*, flow cytometric quantification of neutral lipids after BODIPY staining of cells with or without SorA treatment. Relative fluorescence units were normalized to the WT control (*n* = 8). *F–H*, representative immunoblots (*F*) and densitometric quantification of free GFP/GAPDH (*G*) or full-length GFP-Atg8/GAPDH levels (*H*) of GFP-Atg8–expressing cells with or without treatment with SorA (*n* = 8). *I*, representative micrographs of cells according to *E–H*. PrI served to exclude dead cells from analysis. *Bar*, 5 μm. *Dot plots* show all data points along with the mean (*line*) ± S.D. (*error bar*) ANOVA post hoc Tukey test is shown in *A*, *C*, *E*, *G*, and *H*. *, *p* < 0.05; **, *p* < 0.01; ***, *p* < 0.001 (compared with the respective WT); ##, *p* < 0.01; ###, *p* < 0.001 (comparison as indicated). *RFU*, relative fluorescence units; *a.u.*, arbitrary units.

In WT cells, phosphorylation of Acc1 Ser-1157 (24) is catalyzed by the yeast AMPK orthologue Snf1, which controls *de novo* FA synthesis through inhibition of Acc1 (40). Therefore, we hypothesized that Acc1-dependent modulation of autophagy may represent an important path of Snf1 effects on autophagy during aging. To address this idea, we first tested for epistatic effects of the *acc1^S/A^* mutant and a deletion of *SNF1*. As expected, *SNF1* deletion increased the levels of neutral lipids compared with WT cells and even exceeded lipid levels of the *acc1^S/A^* mutation (Fig. 2*A*). Consistently, Δ*snf1* mutant yeast showed elevated autophagy levels, as assessed by GFP liberation (Fig. 2, *B* and *C*) and microscopic observation of GFP-Atg8–expressing cells (Fig. 2*D*). Importantly, autophagy was not further enhanced by introducing *acc1^S/A^* into the *SNF1* deletion background (Fig. 2, *B–D*), suggesting that Acc1-dependent metabolic adaptations act downstream of AMPK-dependent autophagy regulation in aging yeast. We strengthened this observation by testing whether restoring WT-like activity of Acc1 would prevent the increased autophagy observed in the *SNF1* deletion background. *SNF1* knockout cells treated with SorA displayed neutral lipid levels close to the WT (Fig. 2*E*) and WT-like autophagy levels (Fig. 2, *F–I*). These results parallel our findings on SorA-treated *acc1^S/A^* cells (Fig. 1, *G–I*) and suggest that reducing Acc1 activity reverses elevated autophagy caused by *SNF1* deletion.

As noted above, deletion of *SNF1* had an even stronger effect on lipid levels than the *acc1^S/A^* mutation. Because the *acc1^S/A^* strain carries a single mutation at Ser-1157, this could be explained by the additional Snf1-dependent phosphorylation site present at Ser-659, which presumably remained under the control of Snf1. In contrast to the epistatic effect on autophagy levels, the double mutant *acc1^S/A^*Δ*snf1* showed an additive increase in neutral lipid levels compared with the single mutations (Fig. 2*A*). This adds a layer of complexity to the Acc1-Snf1 metabolic interaction that could be explained by several other targets of the Snf1 kinase (41) or putatively other kinases that may target the mutated Ser-1157 site. To minimize pleiotropic effects of modulating kinase signaling upstream of Acc1, we focused on the *acc1^S/A^* mutant strain as a tool to gain further mechanistic insight into autophagy regulation at the cross-roads of acetyl-CoA and lipid metabolism.

### Enhanced lipogenesis reduces acetate/acetyl-CoA availability concomitant with increased autophagy

**Figure 3.**
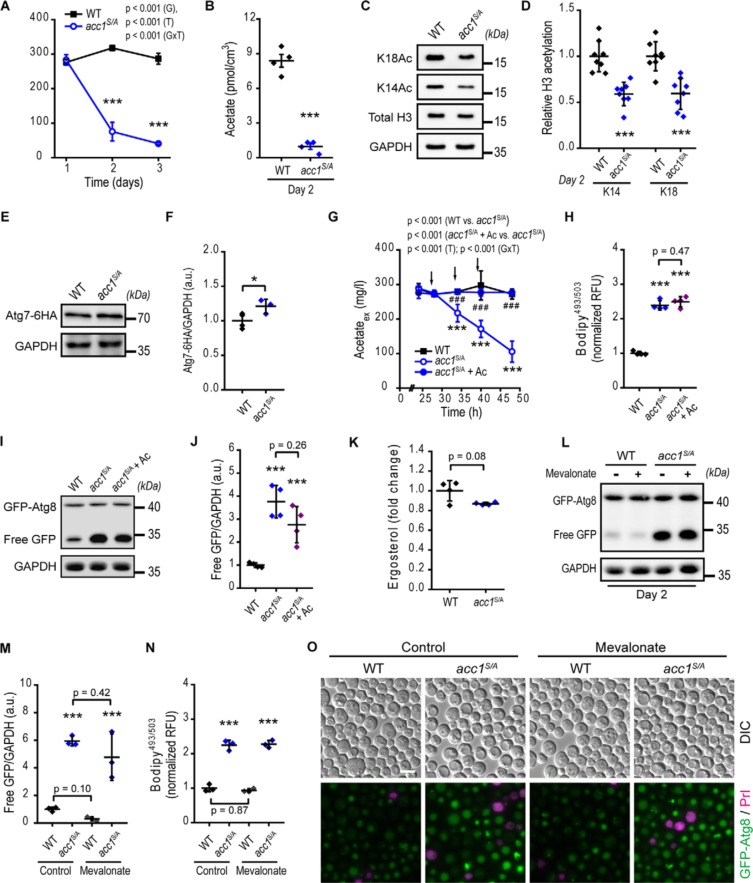
**Acc1 activation stimulates autophagy in concert with changes in acetate/acetyl-CoA metabolism.** WT or *acc1-S1157A* mutant (*acc1^S/A^*) yeast cells with or without supplementation of acetate or mevalonate as indicated were aged to the indicated time points. *A* and *B*, extracellular (*A*) and intracellular (*B*) acetate quantified by NMR from medium samples and cell extracts, respectively (*n* = 4). *C* and *D*, representative immunoblots (*C*) and densitometric quantification (*D*) of histone H3 lysine acetylation after 2 days of aging. Blots were probed with acetylated lysine 14 (*K14Ac*) or 18 (*K18Ac*) specific antibodies or antibodies reacting to total H3 or GAPDH, which served as loading control (*n* = 11–12 for K14Ac, *n* = 8 for K18Ac). *E* and *F*, representative immunoblots (*E*) and quantification (*F*) of C-terminally 6×HA-tagged Atg7 (*Atg7-HA*) using HA-specific antibodies. GAPDH served as loading control (*n* = 3–4). *G* and *H*, extracellular (culture supernatant) acetate (*G*) and flow cytometric quantification of neutral lipids after BODIPY staining (*H*), obtained from cultures receiving three consecutive additions of 100 mg/liter acetate to the medium (+ *Ac*). *Arrows* depict the times of acetate supplementation at 28, 34, and 40 h of incubation (*n* = 4). *I* and *J*, representative immunoblots of GFP-Atg8–expressing cells (*I*) and quantification of free GFP/GAPDH levels (*J*) by densitometry, after 2 days of aging according to *G* and *H* (*n* = 4). *K*, relative ergosterol levels (WT-normalized) quantified from shotgun MS-based lipidomics of lipid cell extracts (*n* = 4). *L–N*, representative immunoblots (*L*), densitometric quantification of free GFP/GAPDH (*M*), and neutral lipids after BODIPY staining (*N*) of GFP-Atg8–expressing cells after 2 days of aging and treatment with mevalonate (*n* = 8). *O*, representative micrographs of 2-day-old GFP-Atg8–expressing cells after mevalonate treatment. Staining with PrI served to exclude dead cells from analysis. *Bar*, 5 μm. *Line graphs* show means ± S.D. (*error bars*). *Dot plots* show all data points along with the mean (*line*) ± S.D. *p* values indicate main effects (genotype (*G*), time (*T*), and interaction between genotype and time (*GxT*)) of a two-way mixed-model ANOVA, with time as repeated measures in *A* and *G*. Welch's *t* test is used in *B*, *D*, *F*, and *K*, and ANOVA post hoc Tukey test is used in *H*, *J*, *M*, and *N*. *, *p* < 0.05; ***, *p* < 0.001 (compared with the WT); ###, *p* < 0.001 (compared with *acc1^S/A^*). *RFU*, relative fluorescence units; *a.u.*, arbitrary units.

We next characterized the metabolic consequences of Acc1 modulation in detail. Acc1*^S/A^* mutation entailed reduced levels of the acetyl-CoA precursor acetate, which, in aging yeast, is indicative of nucleocytosolic acetyl-CoA availability (32). Both extracellular and intracellular levels of acetate were strongly decreased in *acc1^S/A^* compared with WT cells (Fig. 3, *A* and *B*), which may represent the consequence of augmented utilization of acetyl-CoA for FA biosynthesis. The availability of nucleo-cytosolic acetyl-CoA can be rate-limiting for acetylation of histones (42) and represents an important metabolic repressor of autophagy in yeast and mammalian cells (32, 43). Consistently, the reduction in acetate induced by *ACC1* mutation translated into decreased global acetylation of histone H3 at lysine residues 14 and 18 (Fig. 3, *C* and *D*). In agreement with our earlier findings (11, 44), the changes in histone acetylation inversely correlated with the abundance of the autophagy-essential protein Atg7, which increased in *acc1^S/A^* compared with age-matched WT cells after 2 days of chronological aging (Fig. 3, *E* and *F*).

We next aimed to test whether decreased acetate/acetyl-CoA availability or rather the downstream lipidomic changes are causal for changes in autophagy in *acc1^S/A^* cells. To reduce the neutral lipid content without directly affecting Acc1 enzymatic activity, we blocked *de novo* biosynthesis of FAs one step downstream of Acc1 using the FA synthase inhibitor cerulenin (45). Cerulenin successfully reduced the neutral lipid content as well as autophagic activity of *acc1^S/A^* cells to the levels of the WT (Fig. S2, *A–C*). However, cerulenin also restored extracellular acetate levels (Fig. S2*D*), demonstrating the close metabolic interaction of these associated pathways. Interestingly, high concentrations of cerulenin (>0.8 μm) completely blunted the *acc1^S/A^*-dependent increase in neutral lipids and retained lipid levels comparable with those in WT. Under these conditions, however, autophagy was blocked below WT levels with a concomitant increase of acetate, arguing in favor of autophagy repression by elevated levels of acetate and presumably increased acetyl-CoA availability. Next, we restored the decreased acetate levels of *acc1^S/A^* cultures to the concentrations observed for WT cells using three consecutive supplementations of acetate (Fig. 3*G*). However, neutral lipid levels (Fig. 3*H*) as well as autophagy (Fig. 3 (*I* and *J*) and Fig. S1 (*D* and *E*)) remained significantly increased compared with WT cells, demonstrating that reduction of extracellular acetate alone is not sufficient to explain the activation of autophagy in *acc1^S/A^* cells.

Alterations in acetate/acetyl-CoA availability may also translate into changes in mevalonate pathway metabolites (Fig. S3*A*), including the final product of this pathway, ergosterol (46). However, under aging conditions, the single mutation of Ser-1157 (*acc1^S/A^* mutant) almost completely retained WT-like levels of ergosterol (Fig. 3*K*). To rule out a contribution of other mevalonate pathway metabolites to autophagy regulation, we supplemented mevalonate to *acc1^S/A^* cultures, assuming that it would replenish a potential decrease of downstream metabolites. Mevalonate is the product of hydroxymethylglutaryl-CoA (HMG-CoA) reductase, the rate-limiting enzyme of this pathway. Supplementing previously reported concentrations of mevalonate (47) completely reversed cell death induced by the HMG-CoA reductase inhibitor fluvastatin in both aging WT and acc1*^S/A^* cells (Fig. S3*B*) and restored lowered neutral lipid levels of acc1*^S/A^* cells after fluvastatin treatment (Fig. S3*C*). However, mevalonate supplementation did not prevent the strong increase in autophagy in *acc1^S/A^* compared with WT cells, evident from an increased free GFP generation and elevated number and intensity of vacuolar GFP-positive cells (Fig. 3, *L–O*), irrespective of mevalonate treatment, at least after 2 days of aging.

### Inhibition of Acc1 shortens the chronological life span of yeast without alterations in acetate metabolism

We next analyzed whether the reduced LD formation of SorA-treated cells affected general cell viability (48) or the principal capability to form new membranes, a process also crucial for autophagosome formation. For instance, the ability to reinitiate growth when stationary yeast are transferred into fresh medium requires utilization of lipid stores, presumably by providing essential lipid building blocks for membrane biology (31). To compare the growth reinitiation capacity of SorA-treated with that of untreated (control) cells, post-mitotic cultures (*Main culture* after 25 h, Fig. S4*A*) were again transferred into the respective fresh media containing SorA or not (*Shift culture*), and their growth was followed by automated optical density (OD) measurements. This experiment revealed similar initial growth kinetics of SorA-treated compared with untreated control cells (first 4 h of the *Shift culture*, Fig. S4*A*) but expectedly exhibited slowed growth at later time points (>4-h time points of the *Shift culture*, Fig. S4*A*). This suggests that under the selected experimental conditions of the *Main culture*, SorA-treated post-mitotic yeast still contained sufficient lipid stores to maintain general cell viability and generate new functional membranes early during aging.

**Figure 4.**
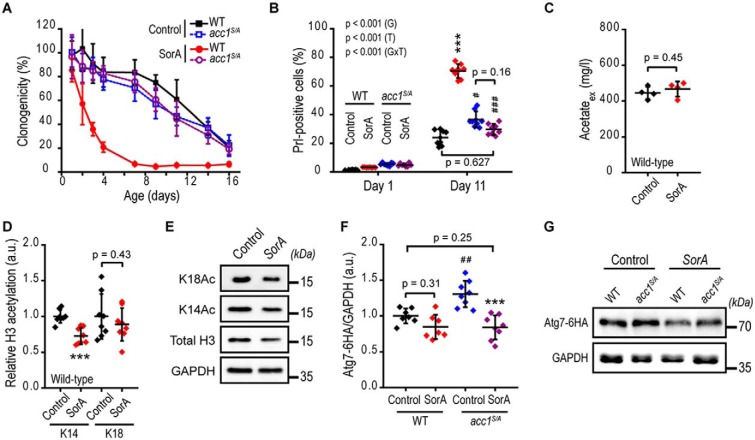
**Inhibition of Acc1 accelerates aging without alterations in acetate/acetyl-CoA metabolism.** WT and *acc1-S1157A* mutant (*acc1^S/A^*) yeast cells with or without SorA treatment were aged until the indicated time points on 2% glucose minimal medium. *A*, survival analysis of chronologically aging yeast assessed by cfu (clonogenicity) (*n* = 8). *B*, cell death assessed by PrI staining (PrI-positive cells) of cells according to *A* and quantified using flow cytometry (*n* = 8). *C*, extracellular acetate quantified enzymatically from culture supernatants (*n* = 4). *D* and *E*, densitometry-based quantification (*D*) and representative immunoblots (*E*) of histone H3 lysine 14 (*K14*) or 18 (*K18*) acetylation in WT cells (with and without SorA treatment) after 2 days of aging (*n* = 8). *F* and *G*, quantification (*F*) of C-terminally 6×HA-tagged Atg7 (*Atg7–6HA*) after 2 days of aging normalized to GAPDH by densitometry of immunoblots representatively shown in *G* (*n* = 7–8). *Line graphs* show means ± S.D. (*error bars*). *Dot plots* show all data points along with the mean (*line*) ± S.D. *p* values indicate main effects (time (*T*), genotype (*G*), and respective interactions (*GxT*)) of a two-way mixed-model ANOVA with time as repeated measures in *B*. ANOVA post hoc Tukey test is used in *F*, and Welch's *t* test is used in *C* and *D*. ***, *p* < 0.001 (compared with the respective untreated control; *Control*); #, *p* < 0.05; ##, *p* < 0.01; ###, *p* < 0.001 (compared with the respective WT). *a.u.*, arbitrary units.

Because functional autophagy is required for stress resistance and cell survival in aging yeast (11, 49), we now tested whether the SorA-induced block in autophagy affected chronological life span. SorA treatment dramatically shortened the life span of WT cells (Fig. 4*A*). Compared with untreated controls, SorA-treated cells displayed reduced clonogenic survival assessed by cfu (Fig. 4*A*) as well as an elevated number of propidium iodide (PrI)-positive cells (Fig. 4*B*), indicative of increased cell death (48). In contrast, SorA failed to reduce the life span of *acc1^S/A^* mutant cells (Fig. 4, *A* and *B*), consistent with our observation that *acc1^S/A^* retained autophagic activity under this condition (Fig. 1, *G–I*). We observed similar effects in the *SNF1* deletion background, which lacks Acc1 inhibition by phosphorylation; the toxic effects of SorA after 2 days of aging were significantly reduced in Δ*snf1* cells, possibly as a result of maintained autophagic levels (Fig. S4*B*). These findings go in line with the allosteric mode of action of SorA to inhibit Acc1 (50) and argue for an Acc1-specific mechanism of SorA-induced cell death and inhibition of autophagy.

As a result of reduced *de novo* production of lipids, inhibition of Acc1 may also affect the FA precursor acetyl-CoA. We reasoned that increased availability of acetyl-CoA may in turn lead to elevated histone acetylation and reduced *ATG7* transcription (32). However, treatment with SorA neither increased the levels of acetate (Fig. 4*C*) nor acetylation of histone H3 at lysine 14 or 18 (Fig. 4, *D* and *E*). It also failed to significantly reduce the protein levels of Atg7 (Fig. 4, *F* and *G*). This goes in line with the observation that GFP-Atg8 is sufficiently expressed and accumulates upon SorA treatment (Fig. 1 (*G* and *H*), *right*). Moreover, Atg7 protein levels remained unchanged in *acc1^S/A^* compared with WT cells when treated with SorA (WT + SorA compared with *acc1^S/A^* + SorA condition; Fig. 4 (*F* and *G*)), despite complete restoration of both SorA-induced depletion of neutral lipids (Fig. 1*F*) and autophagic activity in *acc1^S/A^* cells (Fig. 1, *G–I*). At this point, we again addressed the potential contribution of the mevalonate pathway, because excess acetyl-CoA may also be shuttled into ergosterol biosynthesis. However, ergosterol levels remained constant irrespective of SorA treatment (Fig. S4*C*). In addition, exposure to fluvastatin at subtoxic levels in WT cells (Fig. S4*D*), which should reverse any potential flux into the mevalonate pathway, did not restore autophagy in WT cells treated with SorA, whereas the BODIPY-detectable neutral lipid levels were affected, confirming the effectiveness of the HMG-CoA reductase inhibitor (Fig. S4, *E–G*). In sum, maintenance of physiological rates of autophagy in aging post-mitotic yeast requires functional Acc1, whereas inhibition of Acc1 blocks autophagy and reduces life span independent of upstream acetate/acetyl-CoA or mevalonate metabolism.

### Acc1-dependent regulation of autophagy is independent of triglyceride formation and acts downstream of inositol availability

Constitutive activation of Acc1 enhanced autophagy concomitant with a strong increase in BODIPY-detectable neutral lipids. Neutral lipids are normally stored in LDs, which are mainly composed of steryl esters (SE) and TG. Mass spectrometry–based quantification of SE and TG levels revealed TG as the main neutral lipid class that increased in response to Acc1-mediated lipogenesis (Fig. S5*A*) and decreased upon SorA-mediated inhibition of Acc1 (Fig. S5*B*). In contrast, SE levels were only slightly augmented in response to *acc1^S/A^* mutation (Fig. S5*A*) and were not affected by treatment with SorA (Fig. S5*B*). We therefore asked whether formation of TG was essential for enhanced autophagy in *acc1^S/A^* cells and deleted the two enzymes responsible for TG formation from diacylglycerols (Lro1 and Dga1). Both in WT (containing TG) and Δ*lro1*Δ*dga1* (devoid of TG) yeast strains, modulation of Acc1 activity resulted in comparable autophagic flux changes as indicated by GFP liberation and microscopic observation of GFP-Atg8–expressing cells (Fig. S5, *C–E*). Thus, this argues for a TG-independent mechanism of autophagy regulation by Acc1 metabolic effects.

**Figure 5.**
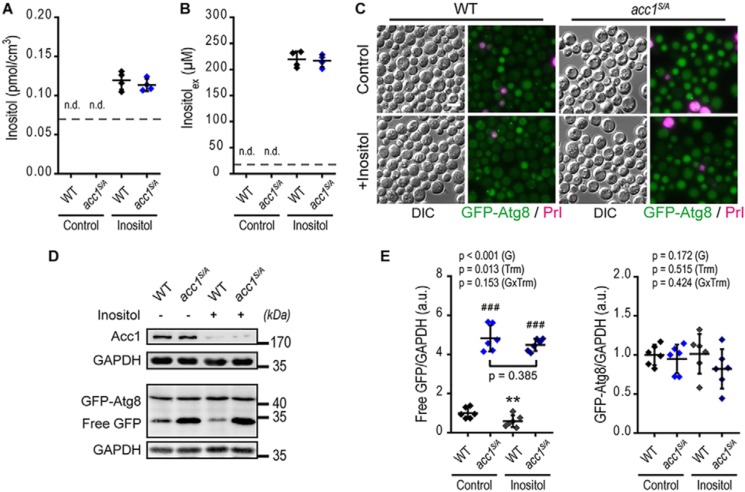
**Acc1 promotes autophagy independent of inositol availability.** WT or *acc1-S1157A* mutant (*acc1^S/A^*) yeast cells were aged on 2% glucose minimal medium and supplemented with extra 750 μm inositol (*Inositol*) or the solvent water (*Control*). *A* and *B*, intracellular (*A*) and extracellular (*B*) inositol quantified by NMR from cell extracts and culture supernatants, respectively, after 2 days of aging. (*n* = 4). *C–E*, representative micrographs (*C*), immunoblots (*D*), and densitometric quantification of free GFP (*E*, *left*) or full-length GFP-Atg8 (*E*, *right*) normalized to GAPDH of GFP-Atg8–expressing cells after 2 days of aging. *Bar*, 5 μm (*n* = 6). *Dot plots* show all data points along with the mean (*line*) ± S.D. (*error bar*). *Dashed lines* depict the lower limit of detection in *A* and *B. p* values indicate main effects (genotype (*G*), treatment (*Trm*), and interaction between genotype and treatment (*GxTrm*)) of a two-way ANOVA in *E*. **, *p* < 0.01 (compared with the respective untreated control; *Control*); ###, *p* < 0.001 (compared with the respective WT). *n.d.*, not detectable. *a.u.*, arbitrary units.

Another reported downstream consequence of constitutive Acc1 activation involves lipid metabolism-dependent alterations in inositol availability. Constitutive Acc1 was shown to cause inositol auxotrophy as a result of Opi1 nuclear translocation and thus suppression of *INO1* transcription (24, 51). Moreover, culturing yeast in inositol-free medium ameliorated the autophagy impairment of LD-deficient yeast subjected to nitrogen starvation, presumably by modulating PL composition (21). We therefore asked whether changes in inositol availability may explain the increased autophagic activity upon Acc1 activation. Notably, our standard experimental conditions contained sufficient amount of inositol in the growth medium to allow WT-like growth of *acc1^S/A^* cultures (Fig. S1*A*). Nevertheless, to rule out autophagy induction by inositol depletion, we supplemented the growth medium with excess amounts of inositol. NMR spectroscopy–based quantification of inositol from both cellular extracts and culture supernatants confirmed similar availability of inositol in *acc1^S/A^* and WT cells after supplementation of inositol (Fig. 5, *A* and *B*). However, excess amounts of inositol did not prevent the increased autophagy observed in *acc1^S/A^* mutant cells, as evident from microscopic analysis of GFP-Atg8 localization (Fig. 5*C*) and from GFP liberation efficiency (Fig. 5, *D* and *E*). However, inositol suppressed autophagy in WT cells along with a reduction in the protein levels of Acc1 (Fig. 5*D*), consistent with a pro-autophagic function of Acc1 and the fact that Acc1 is regulated by the inositol-repressible Ino2/Ino4/Opi1 circuit (23, 52). Autophagy modulation by Acc1, therefore, operates independently of the availability of inositol. Instead, Acc1 appears to function downstream of inositol, as inositol suppresses Acc1 transcription at least under the conditions of cellular aging studied herein.

### Impaired autophagy upon Acc1 inhibition manifests in vacuolar fusion defects

**Figure 6.**
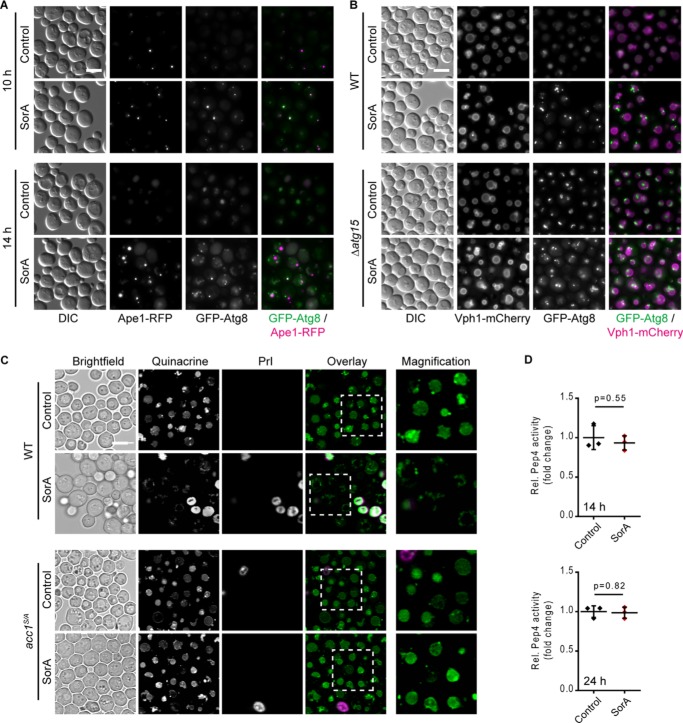
**Acc1 inhibition disrupts autophagosome–vacuole fusion and vacuolar acidification.** WT and *acc1-S1157A* mutant (*acc1^S/A^*) yeast cells were aged until the indicated time points on 2% glucose minimal medium. SorA or the solvent DMSO was applied to cultures 6 h after inoculation. *A*, representative micrographs of WT cells expressing GFP-Atg8 and the PAS marker Ape1-RFP after 10 or 14 h of incubation with SorA. *Bar*, 5 μm. *B*, representative micrographs of WT or *ATG15* deletion mutant (Δ*atg15*) cells expressing GFP-Atg8 and the vacuolar membrane marker Vph1-mCherry after 14 h of incubation with SorA. *Bar*, 5 μm. *C*, representative micrographs of cells stained with the vacuolar acidity indicator quinacrine after 24 h of incubation (day 1). Staining with PrI served to exclude dead cells from analysis. *Bar*, 5 μm. *D*, *in vitro* activity of the vacuolar protease Pep4 in whole-cell extracts at 14 h (*top*) or 24 h (*bottom*) after inoculation. Relative Pep4 activity was normalized to the WT (*n* = 3). *Dot plots* show all data points along with the mean (*line*) ± S.D. (*error bar*). Welch's *t* test is used in *D*.

To characterize the mechanism by which Acc1-dependent metabolic adaptations maintain basal autophagic flux in aging yeast, we first used SorA to test whether key components of the Atg machinery were affected by Acc1 inhibition. The lipidation of Atg8 (Atg8-II), which causes a shift in electrophoretic ability, was unaffected by SorA treatment (Fig. S6*A*), suggesting that the Atg8 lipidation machinery was functional even after Acc1 inhibition. Monitoring Ape1-RFP localization as a marker of the pre-autophagosomal structure (PAS) in the background of autophagy-incompetent *ATG1* deletion strains allows for testing whether GFP-Atg8 properly localizes to the PAS, which is a prerequisite for autophagosome formation (53). Epifluorescence microscopy revealed that GFP-Atg8 co-localized with Ape1-RFP and thus did localize to the PAS (Fig. S6*B*) irrespective of SorA treatment. This suggests that Acc1-dependent regulation of autophagy occurs downstream of Atg1. Similarly, we did not observe SorA-induced GFP-Atg8 puncta formation in the background of a *VPS30* (*ATG6*) deletion strain, again arguing for effects of Acc1 inhibition downstream of Atg6 (Fig. S6*C*). Because early events of the autophagy cascade and autophagic signaling appeared largely unaffected by SorA, we next monitored GFP-Atg8 puncta formation using time-resolved microscopy. At early time points (10-h culture incubation, which is 4-h after SorA application; Fig. 6*A*), GFP-Atg8 mainly co-localized with Ape1-RFP puncta (*i.e.* the PAS) in both control and SorA-treated cells. At later time points, GFP as well as RFP signals were detected within the vacuole in control cells (Fig. 6*A*), suggesting functional autophagy as well as a cytoplasm-to-vacuole-targeting (CVT) pathway, a biosynthetic delivery system that operates via the autophagy machinery. In contrast, from the 10-h time point (4 h after SorA application) onward, SorA-treated cells formed GFP punctate structures that did not co-localize with Ape1-RFP (Fig. 6*A*). This may indicate accumulation of autophagosomes in SorA-treated cells and thus a defect downstream of autophagy initiation and autophagosome formation. Interestingly, Ape1-RFP transport to the vacuole was completely abrogated in SorA-treated cells (Fig. 6*A*), suggesting an impaired CVT pathway. Overall, these data suggest a largely functional Atg machinery involved in the initiation and formation of autophagosomes as well as an active autophagic signaling status even after SorA treatment.

We next addressed later steps in the autophagic cascade by comparing deletion mutants of intravacuolar autophagic body degradation (Δ*atg15*) with the phenotypes observed after SorA treatment. As expected, Δ*atg15* cells displayed intravacuolar GFP-Atg8 fluorescent structures. However, after the addition of SorA, intravacuolar GFP structures disappeared, and Δ*atg15* cells were indistinguishable from WT cells treated with SorA (Fig. 6*B*). This argues for an interference of SorA with the autophagic cascade preceding intravacuolar degradation of autophagic bodies. Strikingly, both epifluorescence (Fig. 6*B*) and confocal (Fig. S6*C*) imaging revealed that the major part (∼75%; Fig. S6*D*) of the GFP punctate structures localized adjacent to or at the vacuolar membrane, which was visualized using mCherry fusion constructs of the vacuolar proton-translocating ATPase (V-ATPase) subunit Vph1. Interestingly, we observed enhanced Vph1-mCherry fluorescence intensities (Fig. S6*B*). Because Vph1 is required for *in vivo* assembly and activity of the V-ATPase (54, 55), we speculated that changes in Vph1-mCherry fluorescence intensity might indicate potential changes in vacuolar acidification. Indeed, staining with quinacrine, a dye that accumulates in acidic compartments (56, 57), revealed that SorA-treated yeast cells lacked vacuolar acidification and accumulated acidic vesicles at the surface of the vacuolar membrane (Fig. 6*C*). At the same time, inhibition of Acc1 did not impair the activity of the master vacuolar protease Pep4 as assayed *in vitro* from cellular protein extracts (Fig. 6*D*). This argues for intact vacuolar proteolytic activity and supports the notion that Acc1 inhibition impacts autophagic flux upstream of vacuolar degradation capacity. Altogether, these findings strongly argue for a vacuolar fusion defect upon Acc1 inhibition that translates into late-stage inhibition of autophagic flux.

### Oleate supplementation mimics Acc1 activation and restores vacuolar fusion defects after Acc1 inhibition

**Figure 7.**
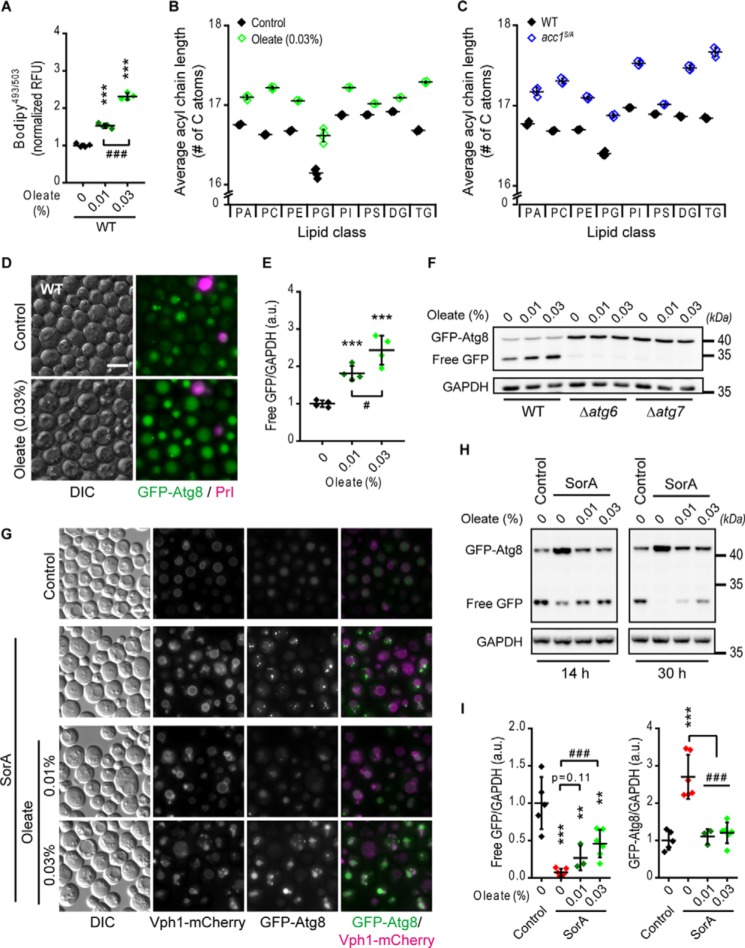
**Supplementation of oleic acid partly mimics *acc1^S/A^* mutation and overrides SorA treatment.** WT yeast cells were supplemented with 0.01 or 0.03% (w/v) oleate or the solvent tergitol (*Control*) 6 h after inoculation and aged until the indicated time points on 2% glucose minimal medium. SorA or the solvent DMSO was applied to cultures 6 h after inoculation where indicated. *A*, flow cytometric quantification of neutral lipids after BODIPY staining of 2-day-old WT cells supplemented with oleate. Relative fluorescence units (*RFU*) were normalized to the WT control (*n* = 4). *B* and *C*, average acyl chain length of different classes of glycerolipids calculated from the lipidomic profiles according to Fig. S7 (*A* and *B*). *B* and *C*, the effects of 0.03% (w/v) oleate treatment (*Oleate*) (*B*) and of the *acc1-S1157A* mutation (*acc1^S/A^*) (*C*) compared with the respective solvent-treated (*Control*) or WT controls, respectively (*n* = 4). *D*, representative micrographs of 2-day-old GFP-Atg8–expressing WT cells supplemented with 0.03% (w/v) oleate. *Bar*, 5 μm. *E*, quantification of free GFP/GAPDH in WT cells from immunoblots representatively shown in *E*. Data were normalized to the solvent control (*n* = 4). *F*, representative immunoblots of 2-day-old GFP-Atg8–expressing WT, Δ*atg6*, or Δ*atg7* cells. *G*, representative micrographs of cells expressing GFP-Atg8 and the vacuolar membrane marker Vph1-mCherry 30 h after inoculation (day 1). *Bar*, 5 μm. *H* and *I*, representative immunoblots (*H*) and densitometric quantification of free GFP/GAPDH (*I*, *left*) or full-length GFP-Atg8/GAPDH levels (*I*, *right*) of GFP-Atg8–expressing cells at 8 or 30 h after inoculation (*n* = 8). *Dot plots* show all data points along with the mean (*line*) ± S.D. (*error bar*). ANOVA post hoc Tukey test is used in *A*, *E*, and *I*. ***, *p* < 0.001 (compared with the respective untreated control); #, *p* < 0.05; ###, *p* < 0.001 (comparison as indicated). *a.u.*, arbitrary units.

A previous study demonstrated *acc1^S/A^* cells to contain an elevated lipid content predominantly composed of C18:1 (oleic acid) acyl chains (24). Consistently, supplementation of external oleate phenocopied consequences of the *acc1-S1157A* mutation, including inositol auxotrophy (24). We therefore asked whether an external supply of oleate would also phenocopy Acc1-dependent changes in lipid profiles and autophagy during post-mitotic aging. Indeed, after 2 days of chronological aging, oleate-supplemented WT cells displayed increased neutral lipid content (Fig. 7*A*) and elicited similar lipidomic changes as observed for *acc1^S/A^* mutant yeast (Fig. 7 (*B* and *C*) and Fig. S7). Approximately 65% of the variance in the lipid species content detected in a lipidomics analysis was shared by *acc1^S/A^* and oleate-treated cells (changes along principal component 1 (PC1) of a principal component analysis; Fig. S7 (*A* and *B*)). Moreover, oleate supplementation increased overall acyl chain length (Fig. 7*B*) but had little effects on the degree of unsaturation (Fig. S7*C*), akin to what was observed for *acc1^S/A^* mutant cells compared with the WT after 2 days of aging (Fig. 7*C* and Fig. S7*D*). Importantly, supplementation of oleate to aging WT yeast was sufficient to boost autophagy (Fig. 7, *D–F*), yet to a lesser degree than detected in *acc1^S/A^* cells (compare with Fig. 1). This induction occurred in an *ATG6-* and *ATG7*-dependent manner, showing that oleate induces autophagy in a canonical fashion during aging (Fig. 7*F* and Fig. S7 (*E* and *F*)).

Finally, we tested whether oleate was capable of reversing the autophagy defect induced by SorA-mediated Acc1 inhibition. The combined treatment of SorA together with 0.01 or 0.03% oleate expectedly prevented the drop in neutral lipid levels observed with SorA alone or even exceeded that of control cultures (Fig. S7*G*). Strikingly, oleate supplementation completely prevented SorA-induced accumulation of GFP-Atg8 puncta at the vacuolar membrane and restored intravacuolar GFP signals (Fig. 7*G*). Consistently, the accumulation of GFP-Atg8 protein levels in SorA-treated yeast as determined via immunoblotting was reversed upon oleate supplementation and dropped again to the levels of control cultures (Fig. 7 (*H* and *I*) and Fig. S7*H*). However, autophagic flux, as indicated by the formation of free GFP, was only partly restored by oleate (Fig. 7 (*H* and *I*) and Fig. S7*H*), arguing for more complex FA requirements to fully restore physiological levels of autophagy. Overall, our results point to a causal role of lipidomic changes in the regulation of age-associated autophagy downstream of Acc1.

## Discussion

In this study, we show that the cytosolic acetyl-CoA carboxylase Acc1 is required for efficient induction of autophagy and maintenance of cell survival in chronologically aging yeast. Constitutive activation of Acc1 through mutation of one of the yeast AMPK (Snf1) phosphorylation sites was sufficient to increase, whereas inhibition of Acc1 blocked, autophagic flux during cellular aging. Additional phosphorylation sites of Snf1 have been described for yeast Acc1 (25). These additional sites may also contribute to regulation of Acc1 activity and autophagy during post-mitotic aging. In fact, forced fatty acid synthesis by mutation of both Snf1-dependent sites together (S659A and S1157A) led to prominent metabolic alterations, including increased fatty acid and general lipid content, and, interestingly, up-regulated genes involved in autophagosome assembly (48). Indeed, deletion of Snf1 strongly induced autophagy under conditions of chronological aging in yeast and thus phenocopied our findings observed by *acc1-S1157A* mutation. With regard to the levels of autophagy, *acc1^S/A^* mutation appears epistatic to *SNF1* deletion, and, more importantly, the increased autophagy mediated by *SNF1* deletion could be reversed by simultaneous inhibition of Acc1. This strongly argues for Acc1 as a crucial target of Snf1-dependent autophagy control under the conditions of post-mitotic aging in yeast. Interestingly, experiments performed with mouse embryonic fibroblasts revealed a similar role of autophagy inhibition by AMPK upon glucose starvation (58). The authors of that study attributed the AMPK-mediated inhibition of autophagic flux in part to ULK1-independent suppression of lysosome acidification (58). This appears in line with our observation that direct inhibition of Acc1, which is an important function of AMPK in both yeast and mammals (59), similarly led to loss of lysosome acidification, accumulation of acidic vesicles at the lysosomal membrane, and loss of autophagic flux likely due to impaired vacuolar fusion of autophagosomes. It is thus tempting to speculate that Acc1-dependent metabolic alterations mechanistically explain AMPK-mediated effects on late-stage autophagy block upon glucose starvation. The precise role of Acc1 in translating the autophagic response to AMPK/Snf1 modulations (41) will be an important question to be addressed in future work: What is the relation of Acc1 to other autophagy-relevant targets of AMPK (*e.g.* ULK1/Atg1), and how does this relate to autophagy signaling by the TOR kinase complexes?

**Figure 8.**
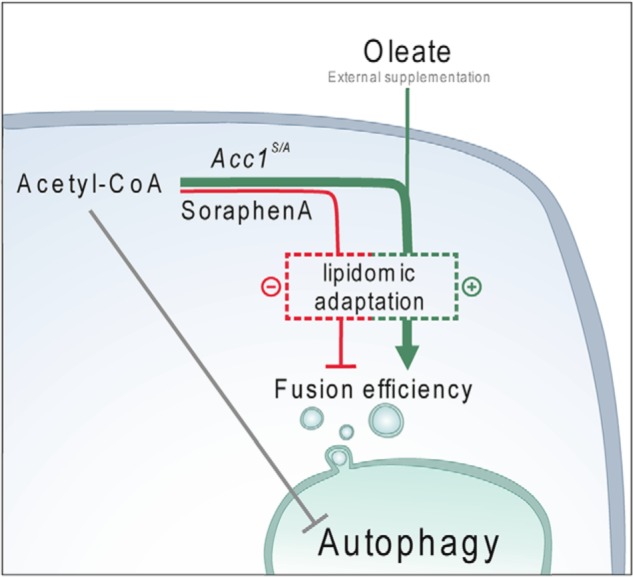
**Metabolic alterations orchestrate Acc1-dependent autophagy.** A *scheme* depicts a working model of how Acc1-dependent lipogenesis drives autophagy in aging cells. Upon Acc1 activation (or after supplementation of oleic acid), increased production of fatty acids and associated lipidomic alterations result in increased autophagic flux. In addition, autophagic activity may be co-regulated by acetate/acetyl-CoA availability upstream of Acc1's metabolic reaction. Mechanistically, Acc1-dependent metabolic consequences provide a cellular lipid profile that facilitates vacuolar fusion capacity and thus allows completion of the autophagic cascade.

Whereas the underlying mechanisms of Acc1-driven autophagy remain to be further clarified, our data suggest distinct metabolic routes of regulation (Fig. 8). On the one hand, we demonstrate that *de novo* FA synthesis is tightly linked to acetate/acetyl-CoA availability under specific conditions. On the other hand, lipidomic alterations are likely to contribute to the autophagic response downstream of Acc1. Acetate/acetyl-CoA availability may affect autophagy through histone acetylation and *ATG* gene transcription as well as via cytosolic protein acetylation (32, 43) and appears relevant in conditions of enhanced Acc1 activity. Along this line, autophagic flux was partially reduced by resupplementation of acetate to *acc1^S/A^* cultures (see Fig. 3*J* and Fig. S1 (*D* and *E*)), indicating that alterations in acetate metabolism may indeed contribute to the full autophagic response after activation of lipogenesis. The mevalonate pathway does not appear to play a major role in Acc1-modulated autophagy during aging, as *acc1^S/A^*-induced activation of autophagy was not prevented by mevalonate supplementation. Consistently, autophagy remained also unaffected by the HMG-CoA reductase inhibitor fluvastatin after SorA treatment.

The autophagy block after inhibition of Acc1 by SorA appears to be solely dependent on downstream metabolic alterations, because acetate levels, the acetylation status of histones, and *ATG* gene expression all remained largely unaffected under this condition. Thus, the route of acetate utilization by *de novo* lipogenesis at normal physiological rates does not appear to be rate-limiting for histone acetylation. This is in contrast to the role of the mitochondrial path of acetate utilization catalyzed by the CoA-transferase Ach1, which causes histone hyperacetylation when impaired (32, 60). Based on the observed metabolic consequences of the *acc1^S/A^* mutant background, it will be interesting to test (although counterintuitive at first sight) whether increased utilization of acetate/acetyl-CoA through enhanced lipogenesis could counteract pathologies associated with protein hyperacetylation, such as maladaptive metabolic responses to alcohol (61) or even chronic high-fat diet (62).

In support of the notion that lipids (downstream of Acc1-dependent lipogenesis) are important as pro-autophagic molecules, oleate supplementation to aging WT yeast mimicked aspects of the activity-enhancing *acc1-S1157A* mutation, including increased neutral lipid content, increased overall acyl chain length of various lipid species, and enhanced autophagic activity. This goes in line with the previously reported shift of the cellular FA content from C16 to C18 (including increased oleate content) upon S1157A mutation of Acc1 (24). Interestingly, supplementation of oleate to growing mammalian cells also induces autophagy in an acute response, yet in a noncanonical way (63). Similarly, yeast lipophagy is activated in an *ATG6*-independent fashion (63). Our data suggest that, during chronological aging, a well-described and physiologically relevant scenario of stationary (post-mitotic) yeast cells (10, 64–66), the supplementation of oleate drives canonical autophagy, as the liberation of GFP from GFP-Atg8 was completely blunted by deletion of *ATG6* or *ATG7*.

The crucial involvement of lipids to facilitate autophagy downstream of Acc1 is underlined by our finding that external oleate (i) prevents the accumulation of GFP-Atg8 punctate structures and (ii) partly restores autophagic flux in cells treated with the Acc1 inhibitor SorA. The fact that SorA treatment produced Ape1-negative GFP-Atg8 puncta at or in close proximity to the vacuolar membrane strongly suggests formation of autophagosomes, which, however, fail to fuse with the vacuole. These results thus argue for late-stage inhibition of autophagy and go hand in hand with the observation that SorA-treated yeast cells fail to induce vacuole acidification, a process interlinked with normal vacuolar function and, for instance, homotypic fusion of vacuoles (67). We have not tested the possibility that, in addition to fusion defects, SorA treatment affects autophagosome formation rate (*i.e.* the number or formation kinetics of autophagosomes). Nevertheless, the accumulation of GFP-Atg8 punctate structures argues for autophagosomes that fail to fuse with the vacuole, resulting in the lack of GFP liberation and therefore impaired autophagic flux. Of note, we observed a considerable (∼25%) fraction of GFP-Atg8 punctate structures apart from Vph1-mCherry–positive membranes (Fig. S7*D*). Therefore, we cannot fully rule out mislocalization of GFP-Atg8 to other compartments upon SorA treatment. For instance, a similar phenotype has been observed upon mutation of the *cis*-Golgi–localized *t*-SNARE protein Sed5 (68). *Sed5-1* mutant cells display defects in vesicular transport due to impaired membrane fusion, resulting in dispersedly distributed Atg8 dots, which raises the possibility of parallels to our observations.

Intriguingly, the precise cellular lipid composition is pivotal for vacuolar fusion capacity and involves various classes of lipids, including DGs, phosphatidic acids, and PI phosphates (69). We therefore propose a lipid-dependent process downstream of Acc1 that facilitates or prevents vacuolar acidification, vacuolar fusion competence, and thus the capacity to promote or block autophagic flux, respectively. However, whether Acc1-dependent lipidomic alterations directly impact vacuolar fusion capacity or, alternatively, act via regulation of vacuolar acidification, which is a prerequisite for vacuolar fusion in yeast (67), remains an open question. Although oleate restored the vacuolar fusion defects, it failed to recover cell survival of SorA-treated yeast upon aging (data not shown), which is consistent with the incomplete restoration of autophagic flux. The lipid requirements that fully complement Acc1 defects are expected to be complex and may require the supplementation of other FA molecules beside oleate. Identifying these additional FAs may uncover the lipidomic needs to maintain full autophagic flux and cell survival in post-mitotic aging.

We extend previous findings that functional lipid storage and associated metabolism are crucial for autophagy regulation in yeast, in particular after nitrogen starvation (18–20). In this context, LDs mainly act by buffering FFAs to limit FFA-induced ER stress and by modulating PL compositions (21). For these reasons, *de novo* synthesis of FA rather appears to block autophagy in LD-deficient yeast (21). Here, we used WT cells, which are fully capable of TG and SE production. In our model, the biosynthesis of FAs seems essential for autophagy and cell survival, at least during chronological aging. Despite increased production of FAs and associated elevation of several distinct lipid species upon activated lipogenesis, Acc1 activity positively correlates with autophagic flux in WT cells likely because neutral lipid storages succeed to buffer excess FFAs, thus limiting FFA-induced stress. We further asked whether TGs themselves were essential molecules to mediate the effects downstream of Acc1 during cellular aging. However, the formation of TGs appeared dispensable to maintain normal autophagy in post-mitotic yeast, as deletion of TG synthesis did not change physiological rates of autophagy or prevent the autophagy-inducing effects of enhancing Acc1 activity. Notably, the ability to form SE could be sufficient to replace some functions of TGs, such as buffering FFA, even in the absence of TG synthesis. Whether TG formation becomes more important in Acc1-induced autophagy under conditions in which cells cannot produce SE and to what extent SE may replace TG function should be addressed in more detail. Despite being prominently affected in their abundance, our data show that TGs are not essential for Acc1-dependent effects on autophagy and thus point to other classes of lipids.

Interestingly, both conditions that we here show to promote autophagy (*i.e.* enhancing Acc1 activity or supplementing oleate) were previously demonstrated to display inositol auxotrophy (25). This raises the question of whether changes in inositol, previously linked to autophagy regulation (70, 71), mediate Acc1- and oleate-dependent autophagy. This would be in line with previous work showing that inositol depletion restores autophagy in LD-deficient cells upon nitrogen starvation (21). Nevertheless, our data and those of Velazquez *et al.* (21) suggest that not inositol itself, but rather its effects on the precise cellular lipid composition, determine autophagic activity. In line with this, we found that inositol supplementation suppressed autophagy along with a reduction in Acc1 protein levels, arguing for Acc1 to act even downstream of inositol.

Oleate supplementation was shown to extend the life span of flies and worms (72, 73). Moreover, previous studies have revealed Acc1 to be required for dietary restriction-induced longevity and starvation resistance in *Drosophila melanogaster* (74). Therefore, it appears plausible that Acc1 represents an important link between the metabolic regulation of autophagy and healthy aging. Autophagy is required for life span extension upon dietary or caloric restriction in several organisms (5, 75, 76). We demonstrate that Acc1 is required for autophagy at least in post-mitotic models of cellular aging. Thus, Acc1-dependent *de novo* lipogenesis could be an important contributor to known health-promoting dietary regimes, which induce or enable the autophagic response. In particular, this may be crucial in the absence of external lipid sources and thus supports the idea that lipogenesis can have important beneficial effects, for instance, in conditions of lipid-associated disorders (77). Acc1 has been demonstrated to regulate Th17 cell–dependent autoimmune responses in mice (78). Moreover, inhibition of Acc1 impaired survival and stress resistance of cancer cells in culture (79, 80) and has been shown to be required for growth and viability of non-small-cell lung cancer *in vivo* in preclinical animal models (81). Additional work will be necessary to investigate a potential role of autophagy under such pathophysiological conditions. Despite the concept that cancer cells exhibit dependence on *de novo* lipogenesis, which may fulfill their lipid biosynthetic demands, autophagy promotion by Acc1 could be of similar importance; given its essential role in cancer cell viability and growth of certain types of cancers or specific tumor contexts, autophagy is similarly suspected to provide tumors with metabolic flexibility (82). Understanding the mechanisms of Acc1-dependent autophagy regulation may therefore help to develop more efficient strategies against age-associated disease.

## Experimental procedures

### Yeast strains and molecular biology

All experiments were carried out in *S. cerevisiae* BY4741 WT yeast and respective mutant strains depicted in Table S1. BY4741 *acc1-S1157A* (*acc1^S/A^)* mutant resulted from crossing and subsequent tetrad dissection of BY4741 *Mat a* WT and a BY4742 *Mat* α strain harboring the *acc1-S1157A* mutation (gift from S. D. Kohlwein (24)). Mutants were selected on YPD plates containing 1 μg/ml soraphen A. EGFP-Atg8 fusion strains were generated by recombination after linear PCR fragment transformation as described previously (83) using a modified pYM-N37 plasmid (pYM-pATG8) as template and primers as described (32), which generates *EGFP-ATG8* fusion constructs under control of the endogenous pATG8 promoter. Similarly, 3HA tagging of Atg8 and introduction of a *MET25* promoter was conducted using pYM-N36 as a template. To generate respective strains with a copper-controlled promotor (*CUP1*) the pYM-N4 plasmid (83) was used as a template. C-terminally 6×HA-tagged *ATG7* strains were generated as described (32). *SNF1* and *ATG15* knockout strains were generated using pFA-hphNT1 (83) and pUG6 plasmids as templates (84), respectively. Deletion of PEP4 was performed in BY4741 WT and Δ*atg7* strains using pUG27 as a template. For all primers used in this study, see Table S2. All mutants were selected on SD −His or YPD plates containing 250 μg/ml nourseothricin (Sigma-Aldrich, catalog no. 74667), 300 μg/ml hygromycin B (Invivogen, catalog no. ant hg 5), or 300 μg/ml G418 (Carl Roth, catalog no. 0239) or as appropriate. Strains containing Ape1-RFP fusions were derived from transformation of the AflII linearized integrative plasmid pPS128 (53) (kindly provided by F. Reggiori and D. J. Klionsky) and selection on SD −Leu plates. For all strains generated herein, at least three different clones were tested for comparable phenotype and, to rule out clonogenic variations, used for the experiments throughout this study.

### Chronological aging experiments and yeast culture conditions

Chronological aging experiments were performed as described using SC 2% glucose medium and culturing at 28 °C under shaking conditions (145 rpm). Clonogenic survival was determined by plating 500 cells on YPD agar and calculating the cfu after 2 days of incubation at 28 °C (64). Cell death was quantified by flow cytometry after PrI staining (11, 85). For inositol supplementation experiments, myoinositol (Sigma, I5125) was added at a final concentration of 750 μm prior to inoculation. Experiments with *SNF1* deletion strains were performed with medium containing 150 μm myoinositol. For soraphen A (37) treatment, cells were inoculated from fresh overnight cultures to an OD of 0.3 at 600 nm (Genesys 10uv photometer, corresponds to ∼6 × 10^6^ cells/ml) and grown to an OD_600_ of ∼1.5 (mid- to late LOG phase) before 0.5 μg/ml soraphen A was added from a stock solution (10 mg/ml in DMSO stored at −20 °C). Final DMSO concentration was 0.25%. Soraphen A was isolated from the myxobacterium *Sorrangium cellulosum* according to published procedures (86). Oleate addition (0.01 or 0.03% (w/v) and added at a culture density of OD_600_ ∼1.5) resulted from a 100-fold stock solution (1 or 3%, respectively, sodium oleate, Sigma-Aldrich, O7501) dissolved in 10% Tergitol® (Type NP-40, Sigma-Aldrich, NP40S) solubilized by briefly heating to 40 °C. Control cultures received DMSO or Tergitol as appropriate. Mevalonate was purchased in its lactone form (Sigma-Aldrich, M4667) and dissolved in 0.1 m NaOH at a concentration of 2 g/ml (stock solution, stored at 4 °C). The stock was added to yeast cultures at a final concentration of 40 mg/ml (47) prior to inoculation. Notably, the pH of the medium was checked after mevalonate was supplemented and found be unchanged. Fluvastatin (Sigma-Aldrich, SML0038) was dissolved in a stock solution (200 mm in DMSO, stored at −20 °C) and added to yeast cultures at a final concentration of 20 μm at a culture density of OD_600_ ∼1.5. This concentration has been previously shown to inhibit yeast HMG-CoA reductase (87). Control cultures received comparable amounts of the solvent DMSO.

For growth kinetics (growth curve) determination, automated optical density measurements were performed using a Bioscreen C^TM^ (Oy Growth Curves Ab Ltd., Finland), equipped with Honeycomb 2 (sterilized) plates, and applying the following settings: temperature, 28 °C; OD measurement every 30 min with 600-nm filter; continuous shaking; stopping shaking 5 s before OD measurement; shaking speed “normal”; shaking amplitude “medium”. Cells from main experimental cultures were used either directly (main cultures) or after shift to fresh medium by washing once with water and inoculating at a density of 0.3 OD (shift cultures). Then cultures were transferred to the Honeycomb plates (300-μl volume), and continuing growth within the Honeycomb plates was monitored for the indicated time intervals.

### Yeast autophagy measurements

Autophagy was assayed either by immunoblotting-based GFP liberation assays (88, 89) (for detailed Western blotting procedure, see below) or monitoring GFP-Atg8 localization using epifluorescence microscopy of strains harboring chromosomally GFP-tagged Atg8 under the control of its endogenous promoter (*pATG8-EGFP-ATG8* strains) as described (32) or with expression driven by the copper-inducible pCUP1 promoter. To induce expression of the pCUP1 promoter, copper sulfate (stock solution: 250 mm in double-distilled H_2_O) was added to the growth medium at final concentrations of 1.6 or 3.1 μm (as indicated) prior to inoculation. Note that the minimal medium used already contained copper sulfate at a concentration of 40 μg/liter (∼0.25 μm), allowing baseline expression of GFP-Atg8 even without the addition of copper. Atg8 lipidation was monitored using strains expressing N-terminally 3×HA-tagged Atg8 strains under the control of the MET25 promoter in the *PEP4* deletion background (88, 89). For microscopic analyses, PrI staining served to exclude dead, potentially autofluorescent cells visualized by standard rhodamine filters. Vacuolar alkaline phosphatase activity of the strain carrying the Pho8ΔN60 variant was assessed according to published methods (39) with modifications as described (32). Briefly, ∼1 × 10^8^ cells were harvested and disrupted with 100 μl of acid-washed glass beads, and protein concentration was measured using a Bio-Rad protein assay. 1 μg of total protein obtained from 3-day-old cultures was used for the assay.

### Protein extracts and immunoblotting

Protein extracts were obtained from chemical cell lysis (90), and an equivalent of 0.2 *A*_600_ (∼1 × 10^7^ cells) were loaded to 12.5%, 7.5% (for Acc1 protein detection), or 15% (for histone acetylation blots) denaturing SDS-polyacrylamide gels. For the Atg8 lipidation assay, 15% SDS-polyacrylamide gels contained 6 m urea, as described before (89, 91). Immunoblotting followed standard protocols with transfer of proteins to either 0.45 or 0.2 μm (for the Atg8 lipidation assay) polyvinylidene difluoride membranes (Bio-Rad) and subsequent probing with antibodies specific for HA (Sigma-Aldrich, H9658, 1:10,000), GFP (Roche Applied Science, catalog no. 11814460001, 1:5,000), Acc1 (Thermo Fisher Scientific, PA5-17564, 1:500), or GAPDH (Thermo Fisher Scientific, MA5-15738, 1:10,000). Signals were recorded digitally using Clarity^TM^ ECL (Bio-Rad) detected by ChemiDoc^TM^ Touch (Bio-Rad) with automatically determined exposure times to avoid signal saturation. Histone acetylation was assessed using acetylated lysine site-specific antibodies (catalog nos. 07-353 (1:5,000) and 07-354 (1:10,000), Upstate, Millipore; catalog no. ab1791 (1:5,000), Abcam). To this end, acid extracts of yeast cells (92), immunoblot procedures, and quantification (expressed as ratios of acetylated lysine per total histone signals) were performed essentially as described, using protein loading amounts previously determined to be in the linear detection range (11). In general, quantification of immunoblotting signals from automatically determined optimal exposures was obtained by using the rectangular volume tool of Image Lab^TM^ software (Bio-Rad) version 5.2.1 with background correction set to “local background.” For comparison of signals from different blots (polyvinylidene difluoride membranes), each blot was normalized to the respective average signals of all samples (individual blots of one experiment always contained the same number of samples per group) before data were pooled for further analysis and the indicated normalizations.

### Measurement of Pep4 activity

Biochemical measurement of Pep4 activity from whole-cell extracts was performed with a fluorometric Cathepsin D activity assay kit (Abcam; ab65302) as described recently (56). In brief, 2 × 10^6^ cells were collected at the indicated time points and resuspended in 200 μl of supplied CD cell lysis buffer. Homogenization was performed with 100 μl of glass beads (500-μm diameter) per sample for 3 × 1 min in a cooled cell disruptor (BioSpec, Mini-Beadbeater-96). Samples were centrifuged for 5 min at 16,000 × *g*, 4 °C, and supernatants were transferred into fresh tubes. Protein concentrations were determined with a Bradford assay (Bio-Rad), and a volume corresponding to 0.1 μg of protein was used for the activity assay according to the manufacturer's protocol. Samples were incubated for 2 h at 28 °C, and the fluorescence signal was analyzed with a Tecan Genios Pro microplate reader (excitation, 328 nm; emission, 460 nm). Of note, lysates treated with 150 μm pepstatin A were used as background control and subtracted from all samples.

### Epifluorescence microscopy

16-Bit images were taken with a Leica DM6B-Z epifluorescence microscope equipped with a Leica-DFC9000GT camera using an HC PL APO ×100/8 NA 1.4 oil lens. The GFP filter set (470_ex_, 525_em_) was used for detection of GFP, and the TXR filter set (560_ex_, 630_em_) was used for detection of RFP or mCherry. The software Leica Application Suite X 3.6.0.20104 was used to capture images with exposure times adjusted to avoid signal saturation and using the autofocus module applied at DIC filter conditions. Raw images were exported as TIF files and further processed using the open-source software Fiji (93). 16-Bit images were converted to 8-bit, and the same display range settings were applied to all images of a given set of samples.

### Automated GFP puncta analysis

16-Bit images were taken with a Leica DM6B-Z epifluorescence microscope equipped with a Leica-DFC9000GT camera using an HC PL APO ×100/8 NA 1.4 oil lens. Exposure was set to 500 ms for GFP (470_ex_, 525_em_) and 600 ms for TXR/mCherry (560_ex_, 630_em_) channels. Raw images were exported as TIF files and analyzed using an automated macro in Fiji (ImageJ 1.52n). First, lower and upper limits of the display range were set to 1,000–50,000 for GFP and 2000–65535 for mCherry, respectively. Total GFP puncta were quantified by down-sampling the GFP image to 8-bit and using the “Find maxima” command (prominence set to 12). Nonvacuolar GFP puncta were quantified by excluding Vph1-mCherry–stained vacuoles, which were segmented by down-sampling the mCherry image to 8-bit and applying automated thresholding (Huang method).

### Confocal microscopy

Specimens were prepared on agar slides for immobilization of yeast cells. Colocalization of endogenously mCherry-tagged Vph1 and GFP-Atg8 was analyzed with a Leica SP5 confocal microscope equipped with a Leica HCX PL APO ×63 NA 1.4 oil immersion objective. The GFP signal was exited at 488 nm and measured between 500 and 530 nm, and mCherry was excited at 555 nm and detected between 590 and 650 nm. *Z*-Stacks were acquired using 63 × 63 × 125 (*x*/*y*/*z*)-nm sampling and processed with the open-source software Fiji (93). Image noise was reduced in *Z*-stacks with three-dimensional Gaussian filtering (*x*σ = *y*σ = *z*σ = 1), followed by background subtraction (rolling ball radius = 50 pixels) and projection of data via the maximum-intensity projection method.

Quinacrine/PrI co-stained cells were visualized with a Zeiss LSM 700 confocal microscope, equipped with a Zeiss Plan-Apochromat 63× NA 1.4 DIC M27 oil immersion objective. Quinacrine was exited at 488 nm and measured between 500 and 530 nm, and PrI was exited at 563 nm and detected between 590 and 650 nm. The Fiji plugin “Iterative Deconvolve 3D” (94) was used for image restoration, which is in part based on the DAMAS algorithm. Thereby, five iterations were applied. Deconvolution of the slightly undersampled image achieved higher contrast of vesicular structures than by the use of Gaussian filtering as described above. All pictures within an experiment were captured and processed in the same way.

### Quinacrine staining

To visualize acidic cellular compartments, quinacrine staining was performed as described recently (56). Therefore, ∼1 × 10^8^ cells were harvested at the indicated time points and washed with 500 μl of YEPD medium, containing 100 mm HEPES (pH 7.6). After centrifugation (16,000 × *g*, 1 min, room temperature) samples were resuspended in 500 μl of YEPD medium containing 100 mm HEPES (pH 7.6) as well as 400 μm quinacrine and were incubated for 10 min at 28 °C and 145 rpm. Subsequently, cells were incubated for 5 min on ice and after centrifugation (16,000 × *g*, 1 min, 4 °C) washed twice in 500 μl of ice-cold HEPES buffer (pH 7.6) containing 2% d-glucose. Afterward, cells were resuspended in the same solution, additionally containing 100 μg/liter PrI, and incubated for 10 min on ice in the dark.

### Yeast neutral lipid measurements

Lipid droplets were visualized, and neutral lipid content was quantified using BODIPY^TM^ 493/503 (Thermo Fisher Scientific, D3922). Approximately 5 × 10^6^ cells were stained in PBS containing 1 μg/ml BODIPY^TM^ 493/503 and 0.1 μg/ml PrI for 15 min in the dark. Cells were harvested via centrifugation at 4,000 rpm for 5 min and washed once with PBS, and the pellet was resuspended in 250 μl of PBS. Staining was visualized by epifluorescence microscopy (standard GFP filters) or quantified by flow cytometry. For quantification, 30,000 cells were analyzed using BD LSR Fortessa (488-nm excitation, 530/30-nm detection), and the mean fluorescence peak area was evaluated using BD FACSDiva 8.0. Dead (PrI-positive) cells (488-nm excitation, 695/40-nm detection) were excluded from analysis.

### Determination of metabolites from yeast culture medium and cell extracts

#### 

##### Metabolites from yeast culture medium

Acetic acid from crude culture supernatants (appropriately diluted with water) was enzymatically measured using the Acetic Acid (Acetate Kinase Manual Format) kit (Megazyme, K-ACETRM) following the manufacturer's microplate protocol.

##### Metabolites from cell extracts

Methanol cell extracts and respective culture supernatants were applied to metabolite analysis by NMR for simultaneous comparison of intracellular and extracellular acetate and inositol.

##### Quenching of cells for metabolite analysis

Culture aliquots of ∼20 OD_600_ equivalents were harvested by filtration using replaceable 0.45-μm sterile filters (Omnipore^TM^ membrane filters, JHWP02500), washed once (on filter) with 5 ml of double-distilled H_2_O, and immediately quenched by deep-freezing the filters in liquid nitrogen. The filtration and washing step was performed in less than 30 s until the freezing step.

##### Generation of methanol cell extracts from quenched cells

Quenched cells (frozen cells on filter) were resuspended in 500 μl of ice cold methanol/H_2_O (2:1) by vortexing thoroughly. 400 μl of the cell suspension were transferred into fresh tube and homogenized with glass beads for 2 × 1 min in a cooled cell disruptor (BioSpec, Mini-Beadbeater-96). The remaining aliquot of intact cells was used for cell count determination by CASY technology (OLS OMNI Life Science, Bremen, Germany), which served to normalize metabolite abundancies after NMR-based profiling. The cell homogenate was then placed on dry ice for 30 min and subsequently stored at −20 °C for 1 h. Next, 200 μl of methanol/H_2_O (2:1) were added prior to centrifugation at 18,000 × *g*, 4 °C for 30 min. 500 μl of the supernatant was transferred to a protein LoBind tube (Eppendorf) and stored at −80 °C prior to NMR metabolic profiling.

##### Reagents for NMR metabolic profiling

Methanol, sodium phosphate, dibasic (Na_2_HPO_4_), sodium hydroxide, hydrochloric acid (32% m/v), and sodium azide (NaN_3_) were obtained from VWR International (Darmstadt, Germany). 3-(Trimethylsilyl)propionic-2,2,3,3-*d*_4_ acid sodium salt (TSP) was obtained from Alfa Aesar (Karlsruhe, Germany). Deuterium oxide (D_2_O) was obtained from Cambridge Isotope Laboratories, Inc. (Tewksbury, MA). Deionized water was purified using an in-house Milli-Q® Advantage water purification system from Millipore (Schwalbach, Germany).

##### NMR metabolic profiling

All chemicals were used without further purification. The phosphate buffer solution (NMR buffer) was prepared by dissolving 5.56 g of anhydrous NaH_2_PO_4_, 0.4 g of TSP, and 0.2 g of NaN_3_, in 500 ml of deionized water and adjusted to pH 7.4 with 1 m NaOH and HCl. The phosphate buffer was lyophilized and redissolved in D_2_O. Supernatants after methanol extraction were dried by vacuum evaporation using a SpeedVac. 500 μl of NMR buffer in D_2_O were added to the dried samples, redissolved, and transferred to 5-mm NMR tubes. Metabolites were measured as described previously (95, 96) and detailed below. All NMR experiments were performed at 310 K on a Bruker Avance III 500-MHz spectrometer equipped with a TXI probe head. The one-dimensional CPMG (Carr-Purcell-Meiboom-Gill) pulse sequence (cpmgpr1d, 512 scans, 73,728 points in F1, 12,019.230-Hz spectral width, 1,024 transients, recycle delay 4 s), with water suppression using presaturation, was used for ^1^H one-dimensional NMR experiments. Bruker Topspin version 3.1 was used for NMR data acquisition. The spectra for all samples were automatically processed (exponential line broadening of 0.3 Hz), phased, and referenced to TSP at 0.0 ppm using Bruker Topspin 3.1 software (Bruker GmbH, Rheinstetten, Germany). Metabolites of interest (acetate, inositol) were quantified using Chenomx NMR Suite Professional 8.2 (Chenomx Inc.) with the existing Chenomx Inc. library and processed one-dimensional CPMG spectra.

### Lipid profiling from yeast cells

#### 

##### Standards for lipid quantification

Synthetic lipid standards were purchased from Avanti Polar Lipids, Inc. (Alabaster, AL). All used solvents were of at least HPLC grade. Stocks of internal standards were stored in glass ampoules at −20 °C until used for the preparation of internal standard mix in 10:3 MTBE/MeOH. 700 μl of internal standard mix contained the following: 1,778 pmol of cholesterol D_7_, 1,040 pmol of cholesterol ester 16:0 D_5_, 521 pmol of 50:0 TG D_5_, 145 pmol of 34:0 DG D_5_, 550 pmol of 25:0 PC, 435 pmol of LPC, 107 pmol of 25:0 PS, 295 pmol 25:0 PE, 85 pmol of 13:0 LPE, 192 pmol of 25:0 PI, 109 pmol of 25:0 PG, 73 pmol of 30:1 Cer, 123 pmol of 25:0 PA, 91 pmol of 13:0 LPA, 32 pmol of 13:0 LPI.

##### Lipid extraction and quantification by shotgun MS

2 OD_600_ units (∼1 × 10^8^ cells) of yeast were homogenized with 0.5-mm zirconia beads in a cooled cell disruptor for 2 × 10 min at 30 Hz in 300 μl of isopropyl alcohol. The whole homogenate was evaporated in a vacuum desiccator to complete dryness. Lipid extraction was performed according to Refs. 97–99. In brief, 700 μl of internal standard mix in 10:3 MTBE/MeOH was added to each sample and vortexed for 1 h at 4 °C. After the addition of 140 μl, H_2_O samples were vortexed for another 15 min. Phase separation was induced by centrifugation at 13,400 rpm for 15 min. The organic phase was transferred to a glass vial and evaporated. Samples were reconstituted in 300 μl of 1:2 MeOH/CHCl_3_. For lipidome and PS measurements, 5 μl of sample were diluted with 95 μl of 4:2:1 isopropyl alcohol/MeOH/CHCl3 + 7.5 mm ammonium formate and 4:1 EtOH/CHCl_3_ + 0.1% trimethylamine, respectively. Mass spectrometric analysis was performed on a Q Exactive instrument (Thermo Fisher Scientific) equipped with a robotic nanoflow ion source TriVersa NanoMate (Advion BioSciences, Ithaca, NY) using nanoelectrospray chips with a diameter of 4.1 μm. The ion source was controlled by the Chipsoft 8.3.1 software (Advion BioSciences). Ionization voltage was +0.96 kV in positive and −0.96 kV in negative mode; back pressure was set at 1.25 p.s.i. in both modes. Samples were analyzed by polarity switching (99). The temperature of the ion transfer capillary was 200 °C; S-lens RF level was set to 50%. Each sample was analyzed for 5.7 min. FT-MS spectra were acquired within the range of *m*/*z* 400–1,000 from 0 to 1.5 min in positive and within the range of m/z 350–1,000 from 4.2 to 5.7 min in negative mode at a mass resolution of *R* at *m*/*z* 200 = 140,000, automated gain control (AGC) of 3 × 10^6^, and a maximal injection time of 3,000 ms. Ergosterol was determined by parallel reaction monitoring FT-MS/MS between 1.5 and 4.2 min. For FT-MS/MS, microscans were set to 1, isolation window to 0.8 Da, normalized collision energy to 12.5%, AGC to 5 × 10^4^, and maximum injection time to 3,000 ms. PS was additionally measured for 1.5 min in negative FT-MS mode with the same parameters as mentioned above. All acquired spectra were filtered by PeakStrainer (https://git.mpi-cbg.de/labShevchenko/PeakStrainer/wikis/home)^7^ (100). Lipids were identified by LipidXplorer software (101). Molecular Fragmentation Query Language (MFQL) queries were compiled for ergosterol, ergosterol esters, PC, LPC, PE, LPE, PI, LPI, Cer, PA, LPA, PG, PS, TG, and DG lipid classes. The identification relied on accurately determined intact lipid masses (mass accuracy better than 5 ppm) and a signal/noise threshold higher than 3 and additional confirmation by targeted MS/MS in a separate measurement for PL and DG species. Lipids identified in the FT-MS measurements were combined in an inclusion list for positive and negative mode and were subjected to targeted MS/MS using these settings: positive and negative FT-MS from 0 to 0.5 min and 11 to 11.5 min, respectively, *R* at *m*/*z* 200 = 140,000, AGC 3 × 106, IT 3,000 ms, *m*/*z* 350–1,000 for positive mode, and *m*/*z* 350–1,500 for negative mode. FT-MS/MS settings were as follows: positive and negative FT-MS from 0.5 to 10.5 min and 11.5 to 35 min, respectively, *R* at *m*/*z* 200 = 140,000, AGC 2 × 104, IT 650 ms, isolation window 0.8 Da, fixed first mass *m*/*z* 120, and NCE 15 and 25% for positive and negative mode, respectively. Lipids were quantified by comparing the isotopically corrected abundances of their molecular ions with the abundances of internal standards of the same lipid class.

##### Lipid data processing

Isotopically corrected abundances of lipid species containing saturated or monounsaturated fatty acyl chains of even length were normalized to the mean abundances of the respective control condition (*i.e.* WT cells or tergitol (vehicle)–treated cells for comparing the *acc1^S/A^* or oleate-treated conditions, respectively) and subjected to principal component analysis using the web-based MetaboAnalyst 4.0 platform (102) without further data normalization or scaling.

### Statistical analysis of the experimental data

Data are presented as *dot plots* with *lines* depicting the mean and *error bars* showing S.D. or as *line graphs* showing means ± S.D. The indicated sample size (see the figure legends) always refers to biological replicates (number of independent cultures per condition). If not otherwise stated, statistical testing was performed using ORIGIN PRO 2016 or IBM SPSS statistics software (version 23). Welch's *t* test (unpaired) and analysis of variance (ANOVA) with Tukey's post hoc tests were used for comparisons between two or multiple groups, respectively. Where appropriate, a two-way ANOVA was applied (independent or mixed design) and was Greenhouse-Geisser–corrected (for mixed-design two-way ANOVA) in the case of sphericity violation as tested by Mauchly's test. The two-way ANOVA was followed by testing simple main effects (*i.e.* multiple comparisons of different levels of each factor that were Tukey-corrected if the factor had more than two levels) in the case of main factor (103) or interaction significance. The reported significance values are always two-sided. Normal distribution of data was confirmed using Shapiro–Wilk's test or judged based on visual inspection of *Q-Q* plots. Homogeneity of variance was tested using Levene's test. Data violating these assumptions were transformed to meet the assumptions. Welch's test (Welch's corrected ANOVA) with Games-Howell–corrected post hoc comparisons were applied whenever homogeneity of variance was an issue.

## Author contributions

A. S. G., A. Z., T. P., S. Schroeder, H. S., O. K., L. L., A. Santiso, D. W., A. A., S. O. L., S. Stryeck, C. K., C. R., S. J. H., B. M., M. W., T. E. performed experiments and analyzed data. R. M. provided soraphen A. D. C.-G., T. M., S. B., K.-U. F., and A. Shevchenko contributed with significant discussions, funding, or interpretation of data. T. E., A. S. G., and S. Schroeder designed and conceived the study. All authors have read and approved the manuscript and their authorship.

## Supplementary Material

Supporting Information

## Footnotes

7 Please note that the JBC is not responsible for the long-term archiving and maintenance of this site or any other third party hosted site.

## Footnotes

6 The abbreviations used are:

LD: lipid droplet
AMPK: AMP-activated kinase
Acc1: acetyl-CoA carboxylase 1
DG: diacylglycerol
PA: phosphatidic acid
PE: phosphatidylethanolamine
PrI: propidium iodide
SorA: soraphen A
TG: triacylglyceride
TSP: 3-(trimethylsilyl)propionic-2,2,3,3-*d*_4_ acid sodium salt
MTBE: methyl *tert*-butyl ether
PC: phosphatidylcholine
PG: phosphatidylglycerol
PI: phosphatidylinositol
PS: phosphatidylserine
FA: fatty acid
FFA: free fatty acid
HMG: hydroxymethylglutaryl
OD: optical density
cfu: colony-forming units
SE: steryl esters
PAS: pre-autophagosomal structure
CVT: cytoplasm-to-vacuole-targeting
V-ATPase: vacuolar proton-translocating ATPase
HA: hemagglutinin
DIC: differential interference contrast
NA: numerical aperture
LPI: lysophosphatidylinositol
LPC: lysophosphatidylcholine
LPE: lysophosphatidylethanolamine
LPA: lysophosphatidic acid
Cer: ceramide
AGC: automatic gain control
ANOVA: analysis of variance
GAPDH: glyceraldehyde-3-phosphate dehydrogenase.
