# Supplementary material for: Acetyl-CoA carboxylase 1–dependent lipogenesis promotes autophagy downstream of AMPK
Source: J Biol Chem. 2019 Jun 17;294(32):12020–39. doi: 10.1074/jbc.RA118.007020 (PMC6690696; doi:10.1074/jbc.RA118.007020)
Supplement: Supporting Information [file supp_294_32_12020__index.html]

Acetyl-CoA carboxylase 1-dependent lipogenesis promotes autophagy downstream of AMPK — Acc1 regulates autophagy — Acetyl-CoA carboxylase 1–dependent lipogenesis promotes autophagy downstream of AMPK — Acc1 regulates autophagy — Supporting Information 

# Acetyl-CoA carboxylase 1–dependent lipogenesis promotes autophagy downstream of AMPK

## Supporting Information

- Supporting Information (to be published online) - Supporting Information containing Tables S1 + S2 as well as Figures S1 to S7
