# Supplementary material for: Acetyl-CoA carboxylase 1–dependent lipogenesis promotes autophagy downstream of AMPK
Source: J Biol Chem. 2019 Jun 17;294(32):12020–39. doi: 10.1074/jbc.RA118.007020 (PMC6690696; doi:10.1074/jbc.RA118.007020)
Supplement: Supporting Information [file supp_RA118.007020_142392_1_supp_348205_pt8vbk.pdf]

Supporting Information

to

Acetyl-CoA carboxylase 1-dependent lipogenesis promotes autophagy downstream of AMPK

Angelina S. Gross[1,*], Andreas Zimmermann[1, 2,*], Tobias Pendl[1,*], Sabrina Schroeder[1, 3], Hannes Schoenlechner[1], Oskar Knittelfelder[4], Laura Lamplmayr[1], Ana Santiso[1], Andreas Aufschnaiter[1, 5], Daniel Waltenstorfer[1], Sandra Ortonobes Lara[1], Sarah Stryeck[6], Christina Kast[1], Christoph Ruckenstuhl[1], Sebastian J. Hofer[1, 3], Birgit Michelitsch[1, 7], Martina Woelflingseder[1], Rolf Müller[8], Didac Carmona-Gutierrez[1], Tobias Madl[3, 6], Sabrina Büttner[1, 5],Kai-Uwe Fröhlich[1], Andrej Shevchenko[4], Tobias Eisenberg[1, 2, 3, #]

[1]Institute of Molecular Biosciences, NAWI Graz, University of Graz, Graz, Austria.
[2]Central Lab Gracia, NAWI Graz, University of Graz, Graz, Austria.
[3]BioTechMed-Graz, Graz, Austria.
[4]Max Planck Institute of Molecular Cell Biology and Genetics, Dresden, Germany.
[5]Department of Molecular Biosciences, The Wenner-Gren Institute, Stockholm University, Stockholm, Sweden.
[6]Gottfried Schatz Research Center for Cell Signaling, Metabolism and Aging, Institute of Molecular Biology & Biochemistry, Medical University of Graz, Graz, Austria.
[7]Division of Plastic, Aesthetic and Reconstructive Surgery, Department of Surgery, Medical University of Graz, Graz, Austria
[8]Helmholtz Institute for Pharmaceutical Research Saarland, Saarbrücken, Germany.

*these authors contributed equally to this work

[#]correspondence to: tobias.eisenberg@uni-graz.at

Content:

Supplemental Table S1 and S2

Supplemental Figures 1-7

Supplemental Table S1

S. cerevisiae yeast strains used in this study. For details on construction of strains refer to the Experimental Procedures

Yeast Strain	Genotype	Origin	Alias
BY4741	*MATa his3Δ-1 leu2Δ-0 met15Δ-0 ura3Δ-0*	*Euroscarf*	
Δ*vps30* (Δ*atg6*)	*BY4741 vps30::kanMX*	*Euroscarf*	
Δ*atg7*	*BY4741 atg7::kanMX*	*Euroscarf*	
Δ*atg7* Δ*pep4*	*BY4741 atg7::kanMX pep4::HIS3*	*This study*	*TEY30*
Δ*pep4*	*BY4741 pep4::HIS3*	*This study*	*TEY31*
Δ*pep4 3HA-Atg8*	*BY4741 pep4::HIS3 pATG8:natNT2-pMet25-3xHA-Atg8*	*This study*	*TEY32*
Δ*atg7* Δ*pep4 3HA-Atg8*	*BY4741 atg7::kanMX pep4::HIS3 pATG8:natNT2-pMet25-3xHA-Atg8*	*This study*	*TEY33*
BY4741 *p8*[1]	*BY4741 pATG8:natNT2-pATG8-EGFP-Atg8*	(32)	*TEY34*
acc1$^{S/A}$ Mat α	*BY4742 (his3Δ-1 leu2Δ-0 lys2Δ-0 ura3Δ-0) acc1$^{Ser1157/Ala}$*	(25)	
acc1$^{S/A}$	*BY4741 acc1$^{Ser1157/Ala}$*	*This study*[2]	*TEY35*
acc1$^{S/A}$ p8	*BY4741 acc1$^{Ser1157/Ala}$ pATG8:natNT2-pATG8-EGFP-Atg8*	*This study*	*TEY36*
Δ*snf1 p8*	*BY4741 snf1::hphNT1 pATG8:natNT2-pATG8-EGFP-Atg8*	*This study*	*TEY37*
Δ*snf1 acc1$^{Ser1157/Ala}$ p8*	*BY4741 acc1$^{Ser1157/Ala}$ snf1::hphNT1 pATG8:natNT2-pATG8-EGFP-Atg8*	*This study*	*TEY38*
BY4741 *Atg7-6HA*	*BY4741 Atg7-6HA-hphNT1*	(32)	*TEY39*
acc1$^{S/A}$ Atg7-6HA	*BY4741 acc1$^{Ser1157/Ala}$ Atg7-6HA-hphNT1*	*This study*	*TEY40*
BY4741 *Pho8ΔN60*	*BY4741 PHO8-URA3-pho8ΔN60*	(32)	*TEY41*
acc1$^{S/A}$ Pho8ΔN60	*BY4741 acc1$^{Ser1157/Ala}$ PHO8-URA3-pho8ΔN60*	*This study*	*TEY42*
BY4741 *pCUP1-EGFP-Atg8*	*BY4741 pATG8:natNT2-pCUP1-EGFP-Atg8*	*This study*	*TEY43*
acc1$^{S/A}$ pCUP1-EGFP-Atg8	*BY4741 acc1$^{Ser1157/Ala}$ pATG8:natNT2-pCUP1-EGFP-Atg8*	*This study*	*TEY44*
BY4741 *p8 Vph1-mCherry*	*BY4741 pATG8:natNT2-pATG8-EGFP-ATG8 Vph1-3mCherry-hphNT1*	(49)	
Δ*atg15 p8 Vph1-mCherry*	*BY4741 atg15::kanMX pATG8:natNT2-pATG8-EGFP-ATG8 Vph1-3mCherry-hphNT1*	*This study*(49)	*TEY45*

[1]*p8* designates strains expressing N-terminally EGFP-tagged Atg8 (EGFP-Atg8) under control of its endogenous promoter (32).
[2]Obtained by crossing *acc1$^{S/A}$ Mat* α (provided by SD Kohlwein) with the BY4741 wild type and subsequent tetrad dissection (see *Methods*).

Supplemental Table S2

PCR primers and templates used for generation of cassettes for mutant generation by gene replacement or tagging by homologous recombination.

**Target**	**Primers**	**PCR template (Reference)**
ATG15 deletion	5'-AACTGATCTAGGCATTACAATTAAAGGAAACAAGGGAAATATTCTATTGACAGCTGAAGCTTCGTACGC-3'<br>5'-GGGCGCATAGGCCCTAAAACAACACTAGGGTCATAATAGATGTATGGGTCGCATAGGCCACTAGTGGATCTG-3'	pUG6 (35)
Control PCR of *ATG15* deletion	5'-CATAGGCTGGGCCATATAC-3'<br>5'-CTGCAGCGAGGAGCCGTAAT-3'	
SNF1 deletion	5'- TTTTTTTTGTAACAAGTTTTGCTACACTCCCTTAATAAAGTCAACCGTACGCTGCAGGTCGAC-3'<br>5'- CATAAAAAAAAGGGAACTTCCATATCATTCTTTTACGTTCCACCATCAATCGATGAATTCGAGCTCG-3'	pFA-hphNT1 (34)
Control PCR of *SNF1* deletion	5'-CGTGATGATGGGACTCGA-3'<br>5'-GTCGACCTGCAGCGTACG-3'	
PEP4 deletion	5'-TCTAGATGGCAGAAAAGGATAGGGCGGAGAAGTAAGAAAAGTTTAGCGCATAGGCCACTAGTGGATCTG-3'<br>5'-ATTTAATCCAAATAAAATTCAAACAAAAACCAAAACTAACCAGCTGAAGCTTCGTACGC-3'	pUG27 (35)
Control PCR of *PEP4* deletion	5'-GCTTGATGTGGTACAACAAG-3'<br>5'-CTGCAGCGAGGAGCCGTAAT-3'	
3HA-tagging of Atg8	5'-CTAATAATTGTAAAGTTGAGAAAATCATAATAAAAATAATTACTA-3'<br>5'-GACTCCGCCTTCCTTTTTTCAAATGGATATTCAGACTTAAATGTA-3'	pYM-N36 (34)
Control PCR of 3HA-tagging of Atg8	5'-AGAGAGCTGGTCAACAGAATCC-3'<br>5'-GTCGACCTGCAGCGTACG-3'	

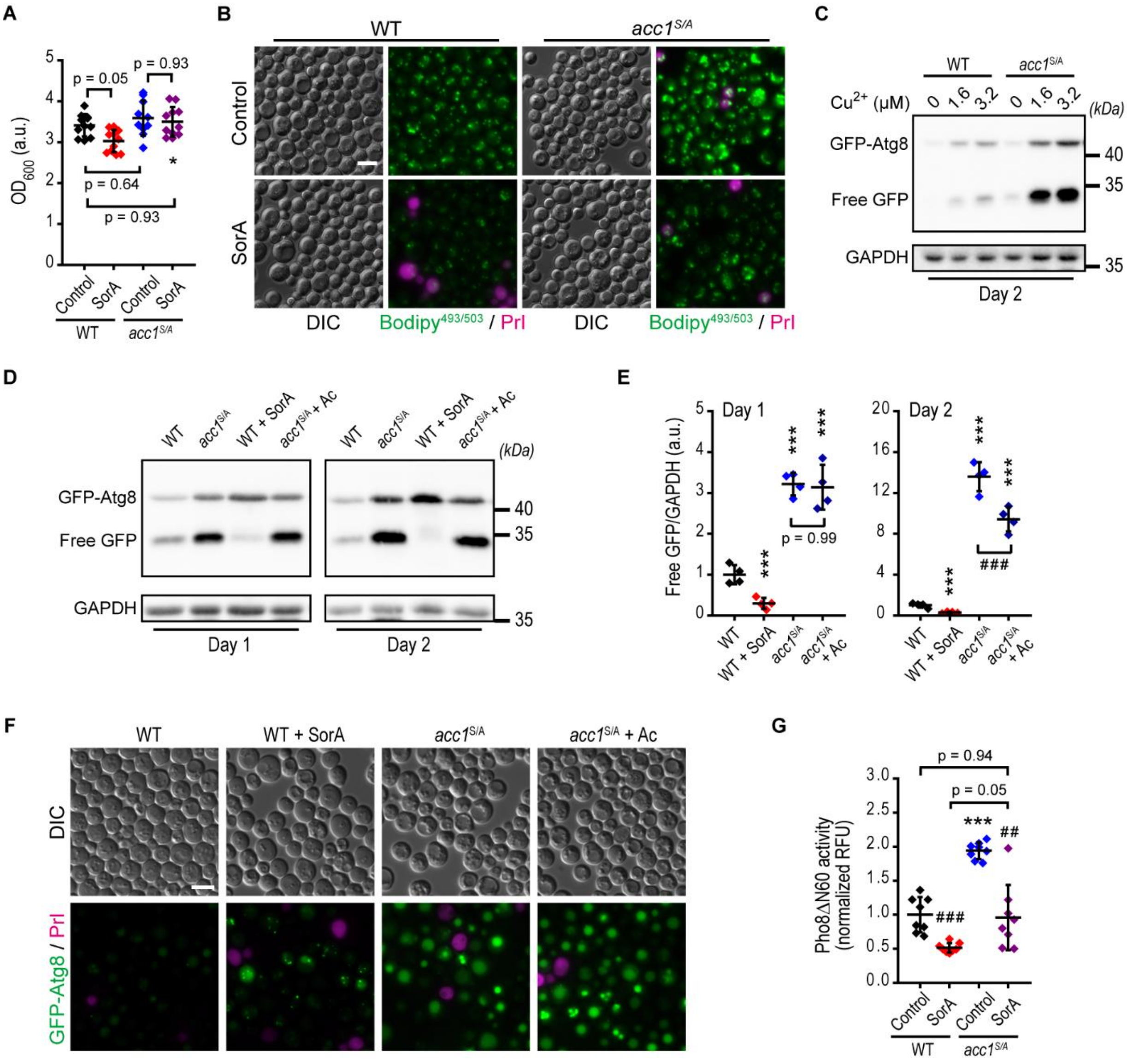

Figure S1 (related to Figure 1)

Modulation of Acc1 activity correlates with alterations in autophagic flux.

(A) Optical density (OD_{600}) obtained using a Tecan™ GeniosPro plate reader of WT or *acc1$^{S/A}$* mutant yeast cultures after 2 days of aging. (n = 10-11)

(B) Representative micrographs of Bodipy stained cells after 24 h (day 1) of incubation. Staining with propidium iodide (PrI) served to exclude dead cells. Bar represents 5 µm.

(C) Representative immunoblots of two-day-old WT or *acc1$^{S/A}$* mutant yeast cells expressing GFP-Atg8 under the control of the Cu^{2+}-inducible CUP1 promoter. Cells were treated with indicated concentrations of Cu_2SO_4 5 h after inoculation.

(Legend continued on next side…)

(D, E) Representative immunoblots (D) and densitometric quantification of free GFP/GAPDH levels (E) of GFP-Atg8-expressing WT or *acc1*$^{S/A}$ mutant yeast cells under the control of the Cu^{2+}-inducible CUP1 promoter at indicated time points. Cells were treated with Soraphen A (SorA) 6 h after inoculation or with three consecutive additions of acetate (+ Ac) following day 1 according to Figure 3G.

(F) Representative micrographs of GFP-Atg8-expressing WT or *acc1*$^{S/A}$ mutant yeast cells under the control of the Cu^{2+}-inducible CUP1 promoter after 48 h (day 2) of incubation according to panels D, E. Staining with propidium iodide (PrI) served to exclude dead cells from analysis. Bar represents 5 µm.

(G) Vacuolar alkaline phosphatase (Pho8ΔN60) activity indicative of autophagic flux at day 3 of aging of WT or *acc1*$^{S/A}$ mutant yeast cells treated with SorA 6 h after inoculation. Genetically engineered strains carrying Pho8 lacking its N-terminal transmembrane domain (Pho8ΔN60) were used. (n = 8)

Dot plots show all data points along with the mean (line) +/- s.d.. ANOVA post-hoc *Tukey* in (A, E), Welch's ANOVA post-hoc *Games-Howell* in (G). $^{*}p < 0.05$, $^{***}p < 0.001$ (compared to the respective WT); $^{\#\#}p < 0.01$, $^{\#\#\#}p < 0.001$ (compared to the respective *Control* or as indicated)

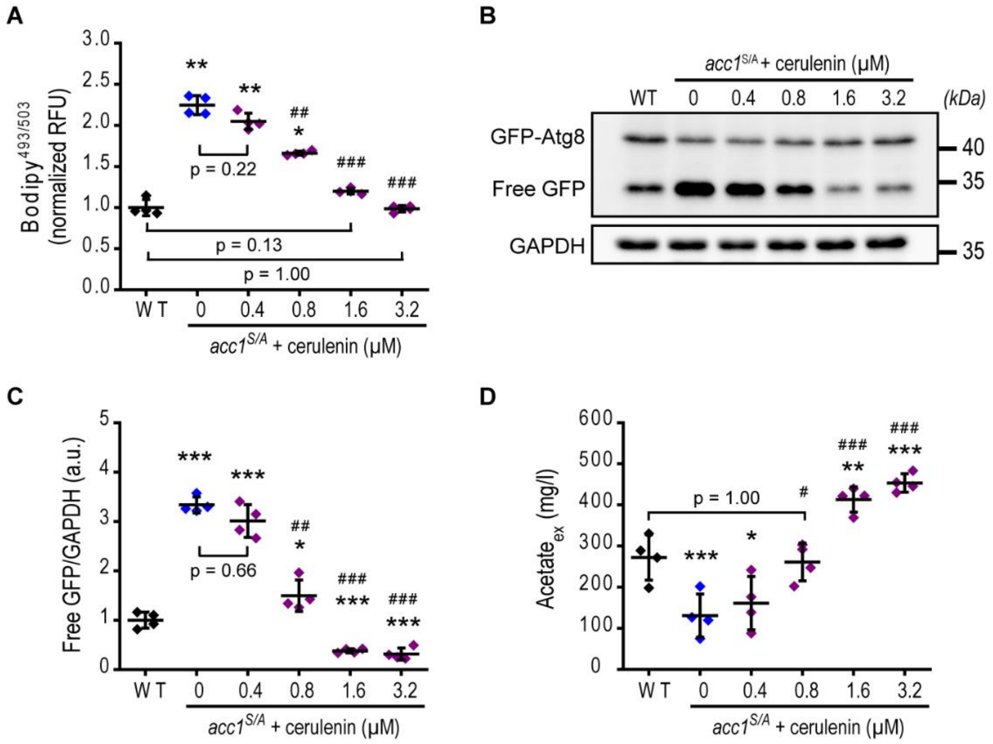

Figure S2 (related to Figure 1 and 3)

FAS inhibition by cerulenin reverses the metabolic consequences and autophagy effects of the *acc1-S/A* mutation

Wild-type (WT) or *acc1*$^{S/A}$ mutant yeast cells were aged on 2% glucose minimal media and treated with the indicated concentration of cerulenin or the solvent DMSO. (n = 4)

(A) Flow cytometric quantification of neutral lipids after Bodipy staining. (n = 4)

(B, C) Representative immunoblot (B) and densitometric quantification of free GFP/GAPDH signals (C) of GFP-Atg8-expressing cells after two days of aging. (n = 4)

(D) Enzymatic determination of extracellular acetate from culture supernatants. (n = 4)

Data were normalized to the WT in panels A, C. Dot plots show all data points along with the mean (line) +/- s.d.. ANOVA post-hoc Tukey in (C, D) and Welch's ANOVA post-hoc Games-Howell in (A). *p < 0.05, **p < 0.01, ***p < 0.001 (compared to the WT); $^{\#}p < 0.05$, $^{\#\#}p < 0.01$, $^{\#\#\#}p < 0.001$ (compared to the respective untreated control).

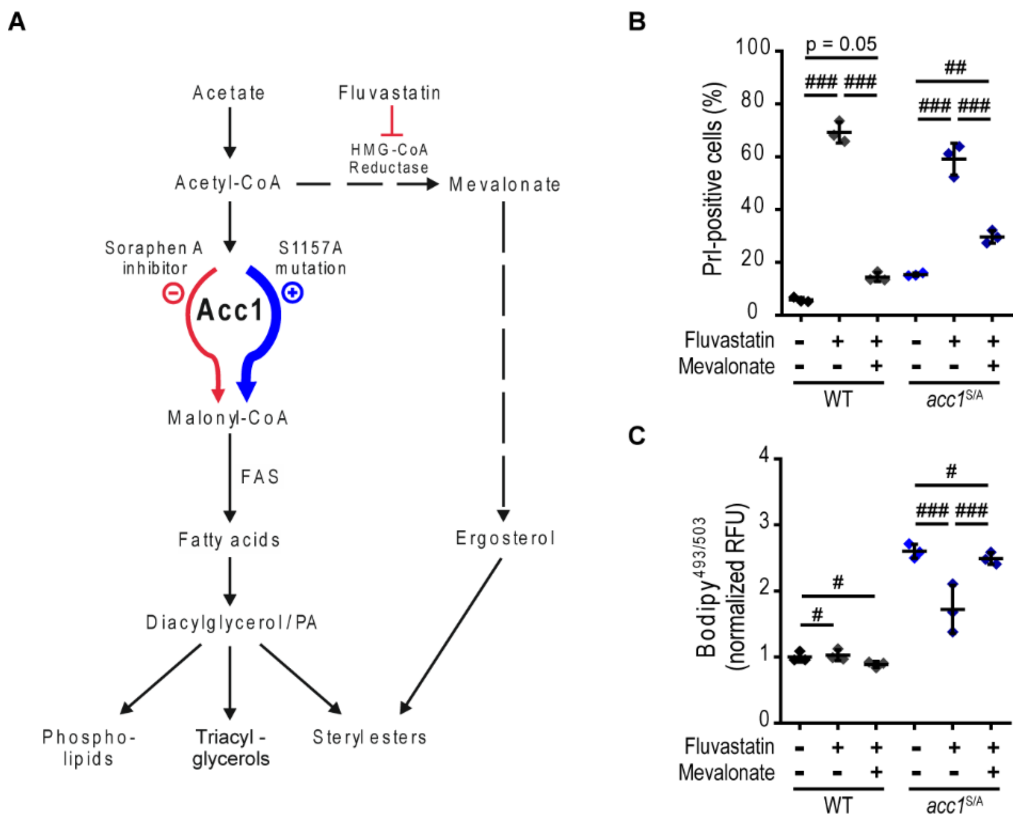

Figure S3 (related to Figure 3)

Mevalonate supplementation prevents cell death upon fluvastatin treatment.

Wild-type (WT) and *acc1*$^{S/A}$ mutant yeast cells were aged on 2% glucose minimal media and treated with 20 µM fluvastatin with or without supplementation of 40 mg/mL mevalonate prior to inoculation.

(A) Scheme depicting the relation of the mevalonate pathway to the Acc1-regulated metabolic reaction. The mevalonate pathway is inhibited by fluvastatin, which blocks the rate-limiting HMG-CoA reductase activity. (PA, phosphatidic acid; FAS, fatty acid synthase; HMG-CoA, hydroxymethylglutaryl coenzyme A)

(B) Cell death assessed by flow cytometric quantification of propidium iodide (PrI) stained cells at day 2 of aging. (n = 3)

(C) Flow cytometric quantification of neutral lipids after Bodipy staining of cells at day 2 of incubation according to panel B. Relative fluorescence units were normalized to the WT control. (n = 3)

Dot plots show all data points along with the mean (line) +/- s.d.. ANOVA post-hoc *Tukey* in (B, C). $^{\#}p < 0.05$, $^{\#\#}p < 0.01$, $^{\#\#\#}p < 0.001$ (comparison as indicated)

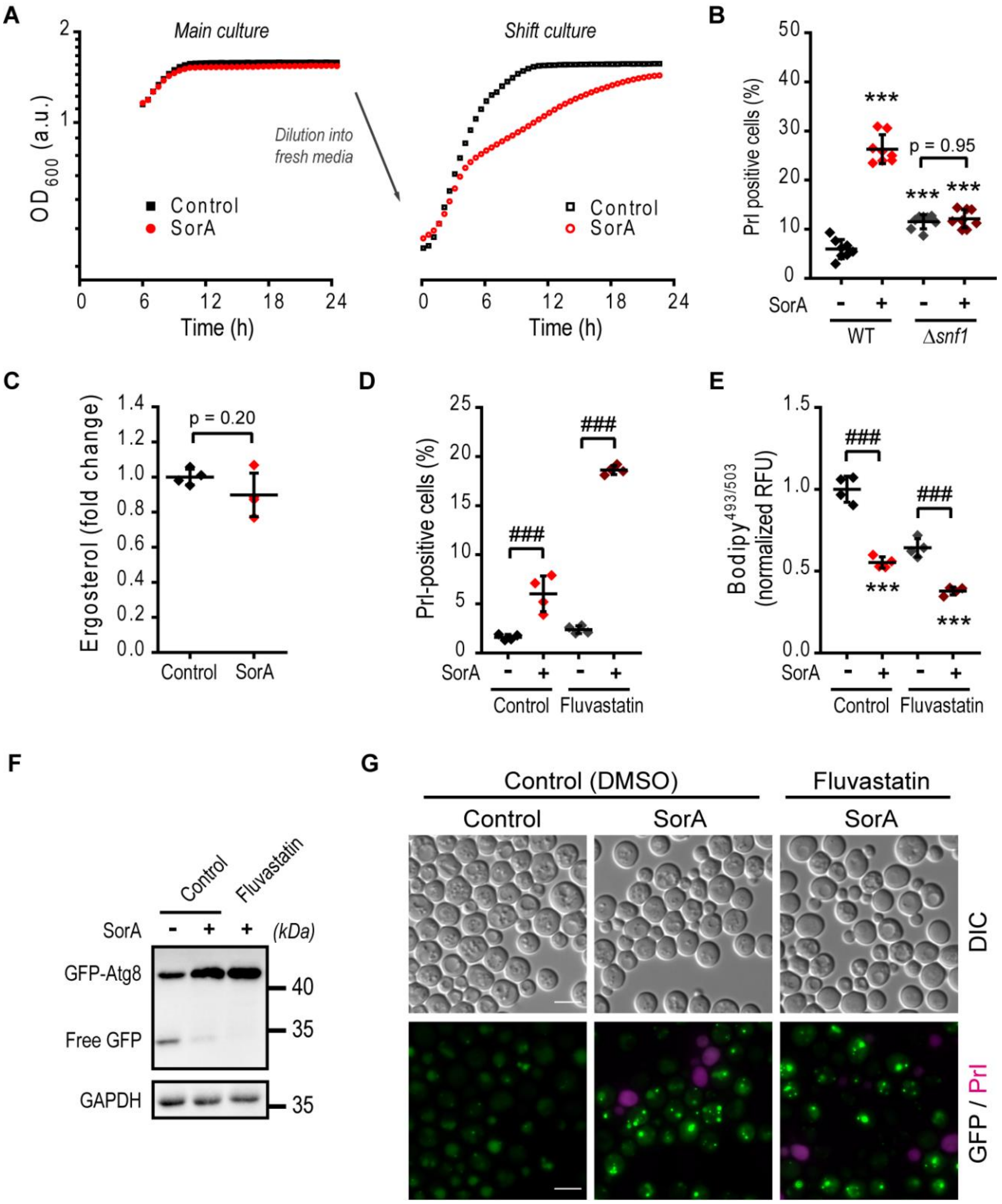

Figure S4 (related to Figure 4)

Inhibition of autophagy by soraphen A does not require the mevalonate pathway.

Wild-type (WT) or Δ*snf1* yeast cells were aged on 2% glucose minimal media and treated with 0.5 µg/ml soraphen A (SorA) or the solvent DMSO (Control) applied 6 h after inoculation.

(A) Growth curves determined by automated optical density (OD) measurements at 600 nm (OD_{600}) using a Bioscreen C™ system.

(Legend continued on next side…)

WT cells from batch cultures according to (Figure 3A) were transferred to Bioscreen microplates 6 h after incubation directly after addition of SorA and residual growth was monitored (*Main culture*). After 25 h of incubation, WT cells from the same batch cultures were shifted into fresh medium of the same condition (with or without soraphen A according to their respective batch culture) and growth in Bioscreen microplates was monitored (*Shift culture*). Data show the means of 4 independent cultures.

(B) Cell death assessed by flow cytometric quantification of propidium iodide (PrI) stained cells after two days of aging. (n = 8)

(C) Relative ergosterol levels (normalized to *Control*) quantified from shotgun MS-based lipidomics of lipid cell extracts. (n = 4)

(D, E) Cell death assessed by flow cytometric quantification of propidium iodide (PrI) staining (D) and neutral lipid levels quantified after Bodipy staining (E) of cells treated with or without 20 µM fluvastatin after 24 h of incubation. (n = 4)

(F) Representative immunoblots of GFP-Atg8 expressing cells according to panels D and E.

(G) Representative micrographs of one-day-old GFP-Atg8-expressing cells according to panels D-F. Staining with propidium iodide (PrI) served to exclude dead cells from analysis. Bars represent 5 µm.

Dot plots show all data points along with the mean (line) +/- s.d.. ANOVA post-hoc *Tukey* in (B, D, E). Welch's *t*-test in C. $^{***}p < 0.001$ (compared to the WT control condition); $^{\#\#\#}p < 0.001$ (comparison as indicated)

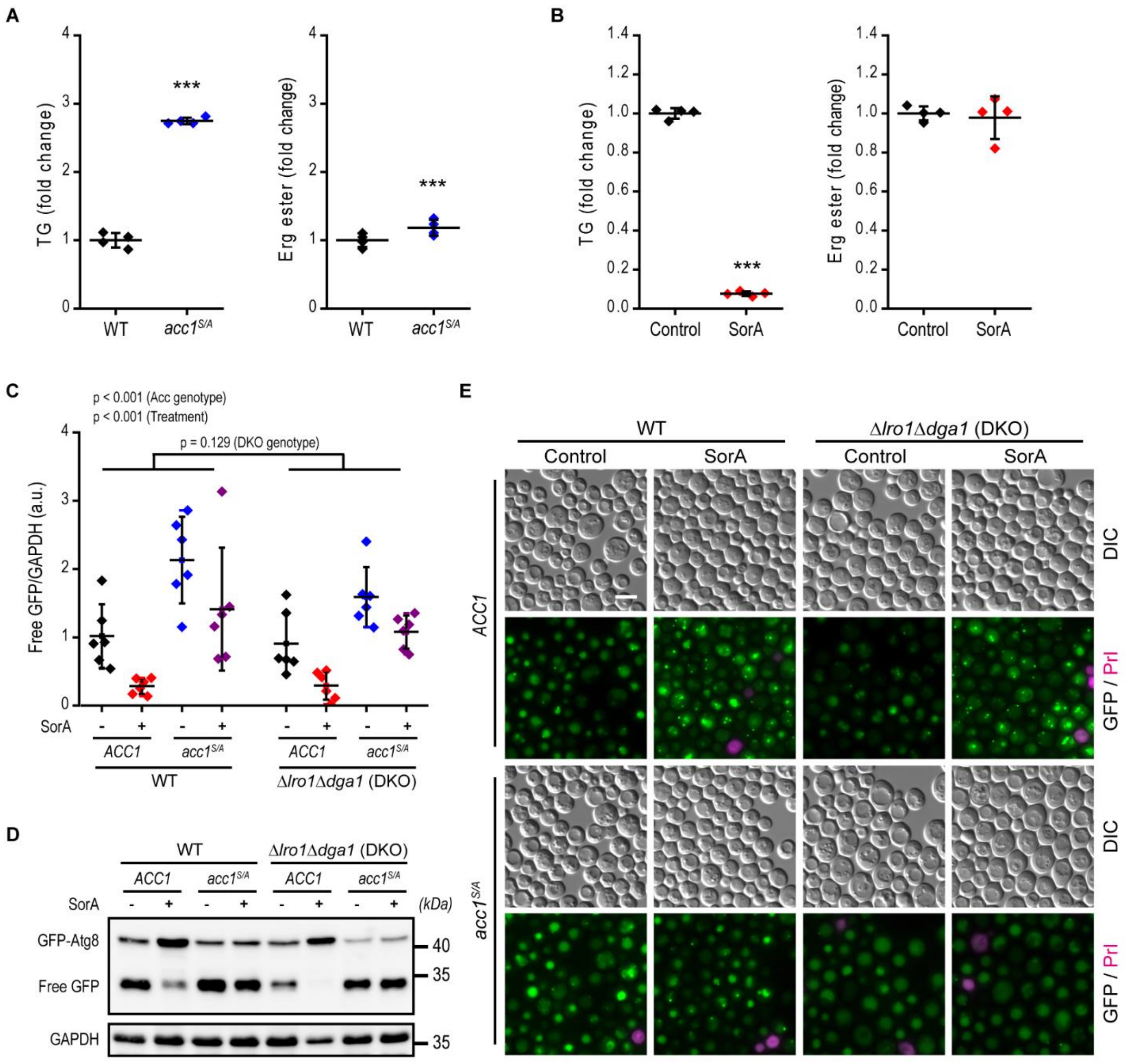

Figure S5 (related to all Figures)

Modulation of Acc1 activity affects autophagy independent of changes in triglycerides.

Wild-type (WT) or Δ*lro1*Δ*dga1* (DKO) combined with or without *acc1*$^{S/A}$ mutation were aged on 2% glucose minimal media and treated with 0.5 µg/ml soraphen A (SorA) as indicated. *ACC1* denotes strains carrying wild-type Acc1. Data were obtained from two-day-old cells.

(A, B) Relative levels of triglycerides (TG, left panel) and ergosterol ester (Erg ester, right panel) as quantified from shotgun MS-based lipidomics of lipid cell extracts. Data were normalized to the WT in (A) and to control cells in (B). (n = 4)

(Legend continued on next side…)

(C, D) Representative immunoblots (D) and densitometric quantification of free GFP/GAPDH (C) of GFP-Atg8-expressing cells. (n = 8)
(E) Representative micrographs of GFP-Atg8-expressing cells. Staining with propidium iodide (PrI) served to exclude dead cells from analysis. Bar represents 5 µm.

Dot plots show all data points along with the mean (line) +/- s.d.. Welch's *t*-test in A, B. *P-values* in (C) indicate the main effects of a three-way ANOVA (*Note: None of the interactions determined within the three-way ANOVA were significant at the p-level of 0.05*). ***$p < 0.001$

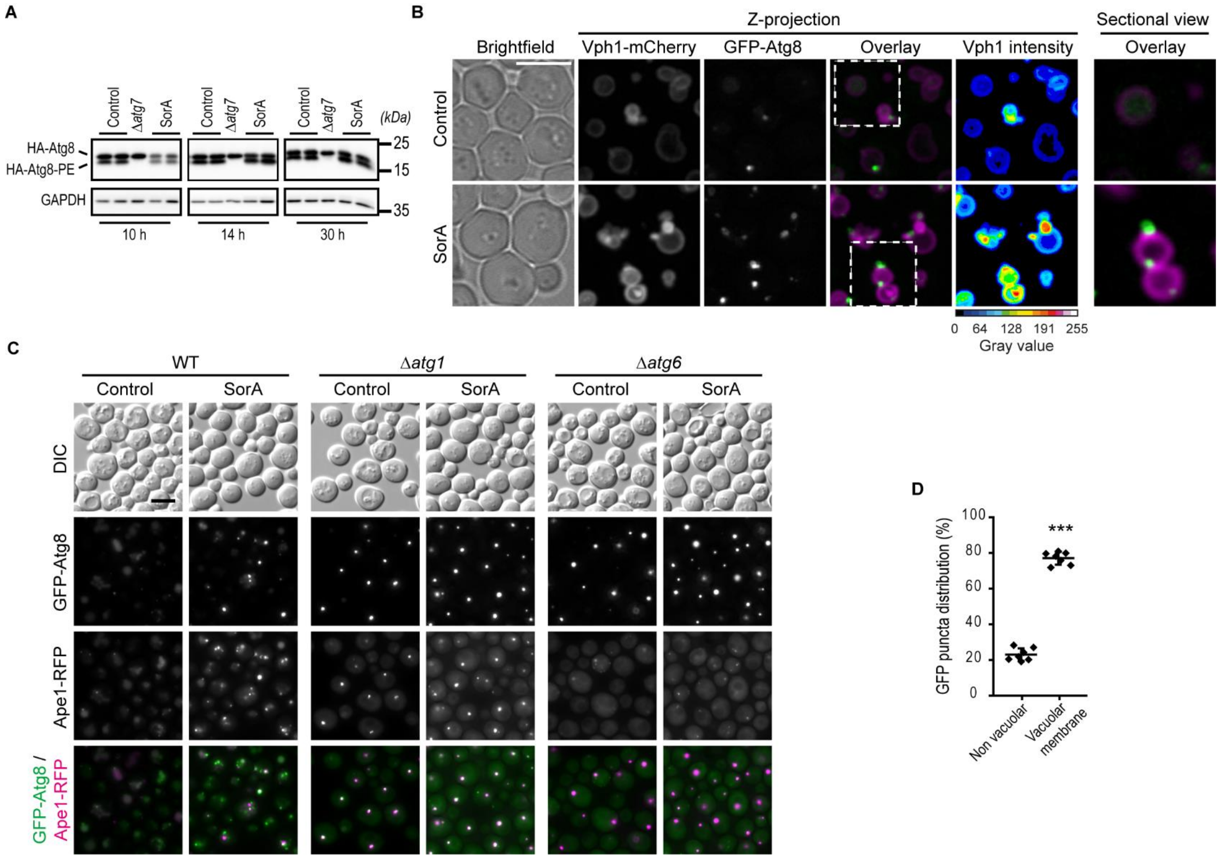

Figure S6 (related to Figure 6)

Acc1 inhibition by soraphen A does not impair the lipidation or PAS-localization of Atg8

(A) Immunoblot analysis of 3xHA-Atg8 expressing Δ*pep4* cells treated with or without soraphen A (SorA) 6 h after inoculation and incubated until the indicated time points. Blots were probed with HA and GAPDH (loading control) recognizing antibodies. 3xHA-Atg8 expressing Δ*atg7* cells served as a negative control lacking the lipidated (HA-Atg8-PE) form of Atg8.

(B) Confocal micrographs of Vph1-mCherry and GFP-Atg8 expressing wild-type cells treated with or without soraphen A (SorA). Z-projection (maximum projection) along with the brightfield image and a selected sectional view (magnification of dashed lined area) is shown. Scale bar represents 5 µm.

(C) Representative micrographs of two-day-old wild-type (WT), Δ*atg1* and Δ*atg6 cells expressing* GFP-Atg8 and Ape1-RFP fusions as a marker of the pre-autophagosomal structure (PAS) site. Cultures were treated with soraphen A (SorA) 6 h after inoculation as indicated. Bar represents 5 µm.

(D) Relative distribution of Vph1-mCherry (vacuolar membrane) localized versus non-vacuolar GFP-Atg8 puncta obtained from automated puncta analysis of micrographs representatively shown in Fig. 6B. (n = 8); ***p < 0.001 (Welch's *t*-test)

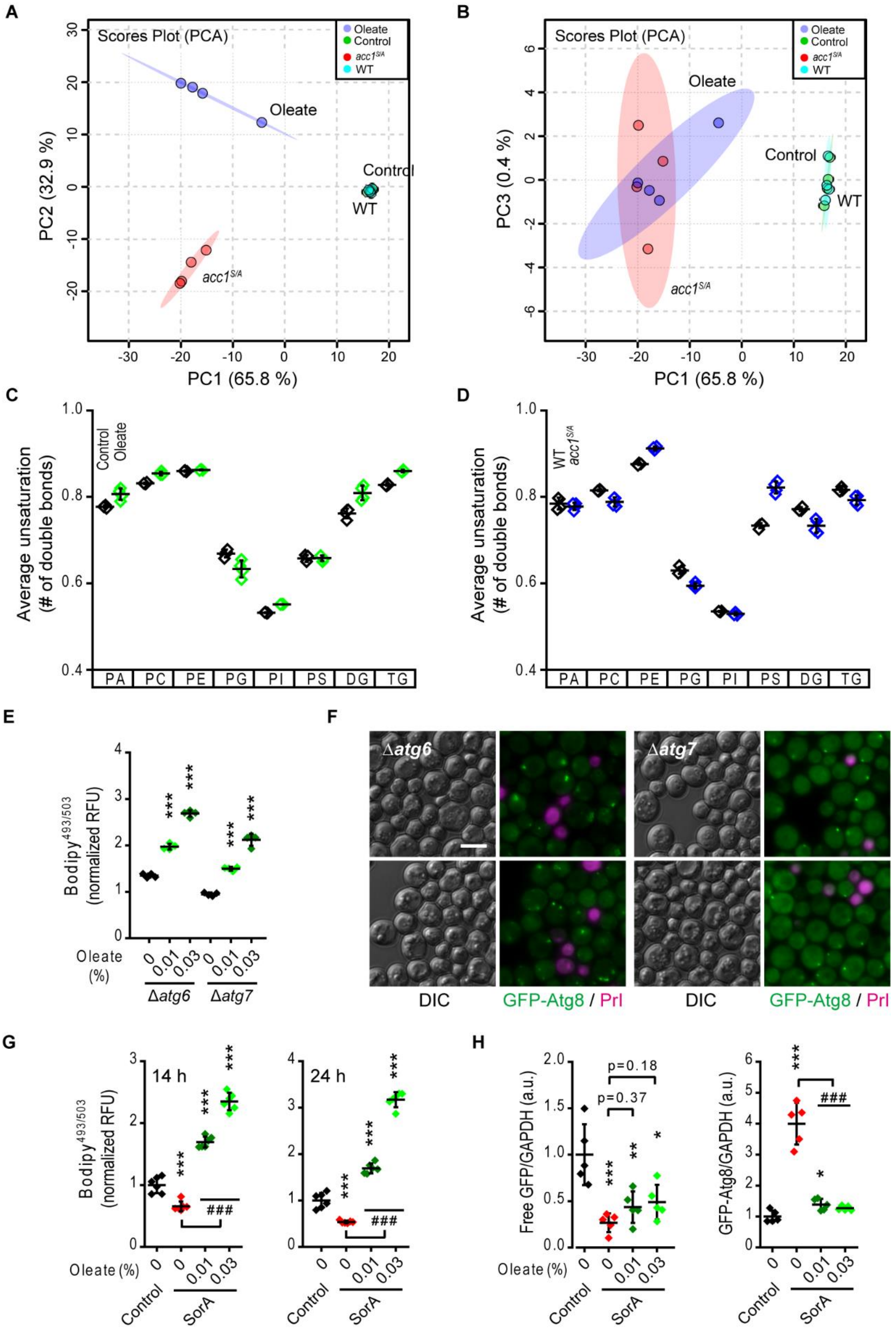

Figure S7 (related to Figure 7) - (*Legend on next side…*)

Supplementation of oleic acid partly mimics the lipidomic adaptations of the *acc1-S/A* mutation

(A, B) Principal component analysis (PCA) of lipid profiles assessed by shotgun mass spectrometry of 2-day-old yeast. The lipid profiles of wild-type (WT) and *acc1*$^{S/A}$ mutant cells are compared to that of WT control cells in the presence of the solvent Tergitol (*Control*) and cells supplemented with 0.03% (w/v) oleate (*Oleate*). Each experimental set was normalized to the mean of its respective control (WT or Control condition) preceding PCA analysis. Ellipsoids show 95% confidence intervals.

(C, D) Average degree of unsaturation of different classes of glycerolipids (as indicated) were calculated from the lipidomic profiles according to (A, B). Panel (C) shows the effects of 0.03% (w/v) oleate treatment (*Oleate*) and (C) of the *acc1-S1157A* mutation (*acc1*$^{S/A}$) compared to the respective untreated (*Control*) or wild-type (*WT*) controls, respectively. (n = 4)

(E) Flow cytometric quantification of neutral lipids after Bodipy staining of two-day-old GFP-Atg8-expressing Δ*atg6* or Δ*atg7* cells supplemented with 0.03% (w/v) oleate or the solvent Tergitol (Control). Relative fluorescence units (RFU) were normalized to the WT control. (n = 4)

(F) Representative micrographs of two-day-old GFP-Atg8-expressing Δ*atg6* or Δ*atg7* cells supplemented with 0.03% (w/v) oleate or the solvent Tergitol (Control). Bar represents 5 µm.

(G) Flow cytometric quantification of neutral lipids after Bodipy staining at indicated time points of SorA treated WT cells with or without supplementation of 0.01% or 0.03% oleate.

(H) Levels of free GFP/GAPDH (left panel) and full length GFP-Atg8/GAPDH (right panel) of immunoblots from GFP-Atg8-expressing cells after 14 h of incubation according to Figure 7H. (n = 5)

Dot plots show all data points along with the mean (line) +/- sd. ANOVA post-hoc *Tukey* in (E, G, H). *$p < 0.05$, **$p < 0.01$, ***$p < 0.001$ (compared to the respective untreated control in E, compared to *Control* in G, H); $^{###}p < 0.001$ (comparison as indicated). *PA, phosphatidic acids; PC, phosphatidylcholines; PE, phosphatidylethanolamines; PG, phosphatidylglycerols; PI, phosphatidylinositols; PS, phosphatidylserines; DG, diglycerides; TG, triglycerides*
